# Vertical accuracy of ASTER, SRTM, and NASADEM digital surface models across Malawi’s diverse topography

**DOI:** 10.1371/journal.pone.0361397

**Published:** 2026-10-29

**Authors:** Kennedy Sones Nazombe, Brighton A. Chunga, Charles Bakolo, Rodrick Mlewa, Godwell Nhamo

**Affiliations:** 1 Department of Land and Water Resources, Faculty of Life Sciences and Natural Resources, Lilongwe University of Agriculture and Natural Resources, Lilongwe, Malawi; 2 Department of Water and Sanitation, Faculty of Environmental Sciences, Mzuzu University, Mzuzu, Malawi; 3 Institute for Sustainability and Corporate Citizenship, University of South Africa, Pretoria, South Africa; 4 Stockholm Environment Institute, World Agroforestry Centre, United Nations Avenue, Gigiri, Nairobi, Kenya; 5 Department of Surveys, Ministry of Lands, Housing and Urban Development, Lilongwe, Malawi; Wallaga University, ETHIOPIA

## Abstract

Digital Surface Models (DSMs) are widely used as important sources of topographic information for earth science and environmental applications. The accuracy of DSMs has direct impacts on several environmental calculations and processes. Therefore, it is imperative to select the most suitable DSM based on the aim and accuracy requirements of each application. However, the use of an appropriate DSM has always been a challenge. This study evaluated the vertical accuracy and performance of the freely available Shuttle Radar Topography Mission (SRTM), NASA DEM (NASADEM), and Advanced Spaceborne Thermal Emission and Reflection Radiometer (ASTER). The DSM elevations were measured against high-precision national geodetic control points. Among other accuracy metrics, we computed Root Mean Square Error (RMSE) and examined the influence of elevation, slope, aspect, curvature, and land cover. At the national scale, SRTM (RMSE of 19.55 m) and NASADEM (RMSE of 19.99 m) showed comparable performance, while ASTER produced a higher RMSE of 22.13 m. However, the principal finding was that DSM accuracy varied substantially across Malawi’s diverse terrain. All the DSMs performed particularly well in the low-lying Lower Shire Valley, with RMSEs of 3.25 m (NASADEM), 4.30 m (SRTM), and 6.91 m (ASTER). Errors increased markedly in the Highlands, where RMSE reached 35.03 m for NASADEM, 33.62 m for SRTM, and 36.94 m for ASTER. In addition, accuracy decreased with increasing elevation and slope, particularly in highland regions and on slopes steeper than 30°. Aspect showed the highest relative importance in the Random Forest analysis. However, the differences in accuracy among aspect classes were comparatively modest, and greater underestimation was observed on north- and west-facing slopes. These findings demonstrate that national-scale accuracy statistics alone do not adequately describe DSM suitability across Malawi’s diverse landscapes. DSM selection should therefore consider the terrain and land-cover characteristics of the intended application area.

## Introduction

Digital Elevation Model (DEM) is a general term for a model that represents the elevations or heights of a topographic surface as a georeferenced grid of points or areas, covering the Earth’s surface or other solid celestial bodies [1]. A DEM generally refers to a georeferenced raster or grid representing surface elevation, while a Digital Surface Model (DSM) is a type of elevation model that represents the elevation of the Earth’s surface, including all objects on it [2]. DSMs provide fundamental information about the Earth’s surface and are indispensable in a wide array of geospatial applications. DSMs have wide applications, including in environmental hazard assessment [3], bathymetric mapping [4], surface water body detection [5], 3D building modelling [6], forest structure mapping [7], flood and water flow modelling [8,9], infrastructure planning [10], and geomorphological mapping [11].

Access to elevation data has increased globally over the years, with the freely available DEMs and DSMs. Advanced Spaceborne Thermal Emission and Reflection Radiometer (ASTER), Shuttle Radar Topography Mission (SRTM), NASA DEM (NASADEM), TanDEM-X 90 m DEM, and Multi-Error-Removed Improved-Terrain DEM (MERIT DEM) are examples of the most common global DSMs in the public domain. Despite their widespread use, the accuracy and applicability of these products are of great concern. Studies have shown that these global DSMs are subjected to varying degrees of vertical inaccuracies [12–14]. The vertical accuracies are influenced by terrain complexity, vegetation, land cover, and sensor-specific characteristics [15–17]. Studies continue to highlight the importance of accounting for topographic effects when processing remotely sensed products, as terrain-induced distortions can substantially influence derived geospatial information [18].

The relative and absolute accuracy of global DSMs (SRTM, NASADEM, and ASTER) has been studied at different scales [12,19,20]. The general finding of the many studies has been that the accuracy of DSMs varies significantly with region and topographic conditions [12,14,21]. There is a consistent observation that SRTM and NASADEM mostly provide more reliable elevation estimates than ASTER [12,14,21]. In addition, the performance of SRTM and NASADEM deteriorates in steep and rugged terrain [14]. Land cover further influences DEM performance [22]. Forested and densely vegetated regions are prone to greater errors due to canopy effects, because the deployed sensors are unable to penetrate the forest cover fully [23]. These findings show that DEM and DSM accuracy is context-dependent and cannot be generalised across all landscapes.

Global insights about the performance of various DEMs are valuable. DEM errors vary from one region to another since they depend on topographic conditions [24]. Malawi’s physiography is very diverse. It encompasses the low-lying Shire Valley, extensive lakeshore plains, mid-elevation plateaus, and rugged highlands that exceed 2,500 m above sea level. This variability creates substantial challenges for DEM accuracy, as errors tend to vary with topography and land cover [22]. Malawi’s diverse land cover includes cropland, grassland, forest reserves, built-up areas, and wetlands [25]. This adds extra complexity. It is well established that vegetation density and surface roughness introduce DEM biases [26]. The need for accurate elevation data to support Malawi’s national development priorities cannot be underestimated. Researchers, practitioners, and policymakers in Malawi often rely on DEMs without clear local evidence of their accuracy in different topographic contexts. Open-source global DEMs/DSM have been used in Malawi in flood and hydrological modelling [27,28], landslide hazard assessment and mapping [29], geophysical mapping [30], and land suitability mapping [31] without local validation of these products. It is thus essential to undertake a systematic, context-specific performance assessment of DEM products in Malawi to help users select appropriate DEM products for different applications. Such an assessment can also inform strategies to mitigate DEM errors and enhance the reliability of geospatial analyses.

This study addresses an important gap in the assessment of DSMs by demonstrating that their vertical accuracy varies substantially with terrain and landscape characteristics and therefore cannot be adequately represented by a single national accuracy statistic. Beyond assessing overall vertical accuracy, the study examines how elevation errors vary with elevation, slope, aspect, curvature, land cover, and physiographic region. This provides insight into the specific landscape conditions under which DSM performance deteriorates. This comprehensive, terrain-stratified assessment provides empirical evidence to inform the selection and application of freely available global DSMs in data-scarce environments. DSM accuracy has implications for hydrological modelling, erosion assessment, flood mapping, land-use planning, and infrastructure development.

Therefore, the present study evaluated how well SRTM, ASTER, and NASADEM estimate elevations in Malawi. To the best of our knowledge, the performance of the three global DSMs has not been evaluated in Malawi. The present study aims to address this gap by further examining how DSM errors vary with topographic factors (elevation, slope, aspect, curvature, and land cover). The findings of this study provide essential evidence on the suitability of global DSMs in a data-scarce, topographically complex country. It also offers practical guidance for researchers, planners, and policymakers in Malawi when using these products. It also contributes to the broader body of knowledge on DSM accuracy in diverse landscapes.

## Materials and methods

### Study area

Malawi is in southeastern Africa along the Great Rift Valley and covers an area of about 118,480 km^2^ (Fig 1). It is bordered by Mozambique to the south, southwest, and east, Tanzania to the north and northeast, and Zambia to the west. The topography of Malawi is defined by the Great Rift Valley depression, which forms Lake Malawi (covering about 20% of the country) and the Shire River. Surrounding the depression are elevated plateaus, including the Nyika Plateau in the north and the Shire Highlands in the south. The mountains are marked by the isolated peak of Mulanje, which rises to approximately 3,000 m above sea level. This is not only the highest peak in Malawi but also in south-central Africa. In addition, Malawi’s landscape ranges from flat plains along the lakeshore and the southern Shire Valley to sharply rising, rugged mountain ranges. Thus, Malawi is subdivided into four main physiographic regions, namely, the lower Shire Valley (<200 m above sea level), the lake shore plain and the middle and upper Shire (200 m to 760 m), the mid-elevation plain (760 m to 1300 m above sea level), and the Highlands (>1300 m above sea level) [32].

**Fig 1:**
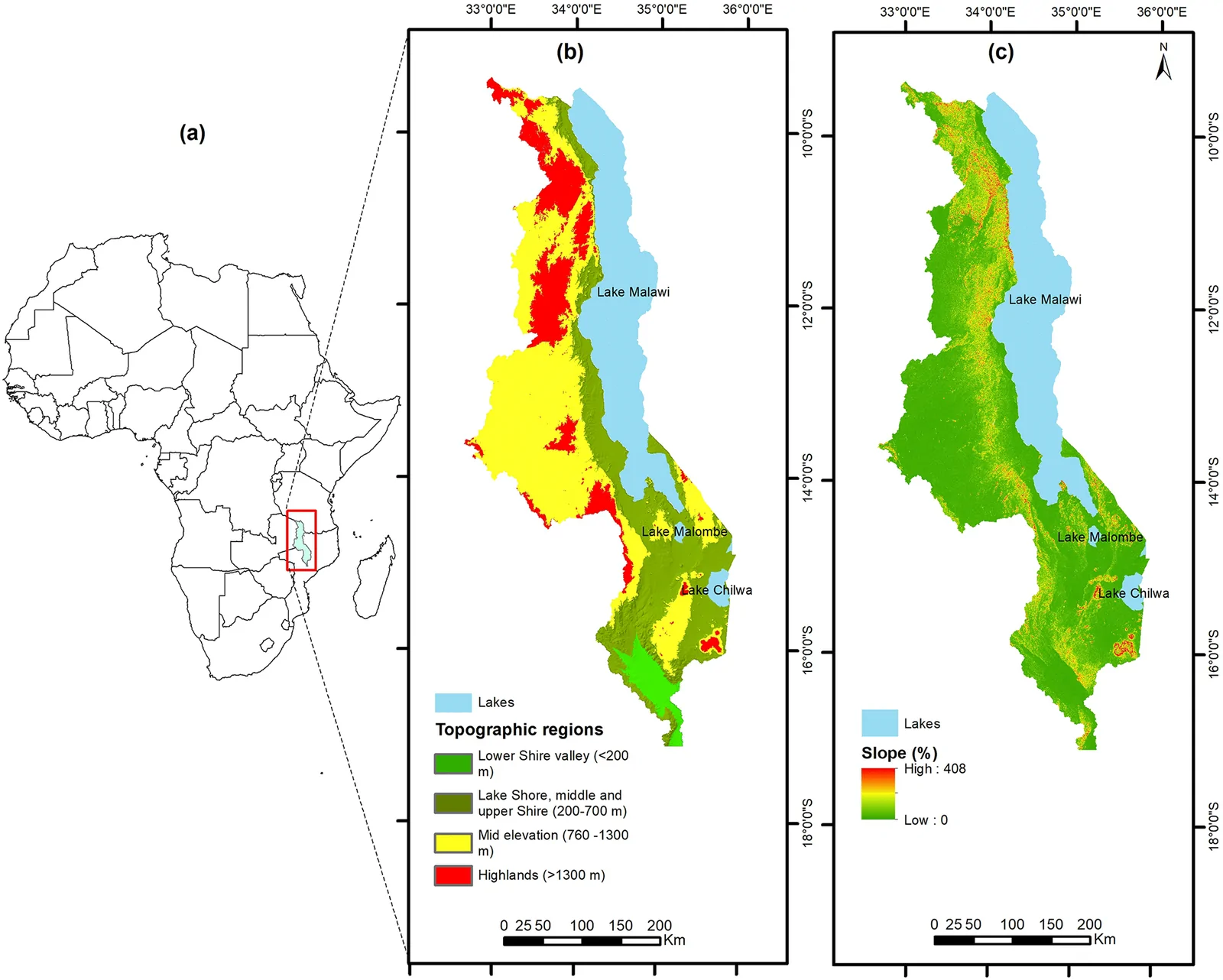
(a) Map of Africa showing the location of Malawi (Data source: Natural Earth http://www.naturalearthdata.com/). (b) The elevation map, which classifies Malawi into four distinct topographic regions, showing topographical variations; (c) Slope map of Malawi derived from SRTM (Data Source: NASA Earthdata).

### Data sources

#### The studied DSMs.

In this study, we assessed the accuracy of three freely available global DSM datasets: ASTER, SRTM, and NASADEM. The basic details of the DSMs assessed are provided in Table 1.

**Table 1:** Comparison of key characteristics of three global Digital Surface Models (DSMs): ASTER GDEM V3, SRTM 1 Arc-second, and NASADEM.

Aspect	ASTER GDEM V3	SRTM	NASADEM
Producer	NASA + METI (Japan)	NASA (Shuttle Radar Topography Mission, 2000)	NASA JPL (reprocessed SRTM, 2020)
Production Method	Optical stereophotogrammetry from ASTER (Terra satellite) using near-infrared stereo pairs	Radar interferometry (InSAR) from two radar antennas on the Space Shuttle mission	Reprocessed SRTM radar data + auxiliary datasets (ASTER GDEM V3, ICESat) with improved geodetic alignment and void filling
Data Acquisition	2000–2013 (multi-temporal)	Feb 2000 (11-day mission)	Based on SRTM 2000, reprocessed in 2020
Coverage	83°N to 83°S (near global)	60°N to 56°S (~80% global land)	Same as SRTM (60°N to 56°S)
Resolution	1 arc-second (~30 m)	1 arc-second (~30 m, global since 2014)	1 arc-second (~30 m)

### The ASTER GDEM

The Advanced Spaceborne Thermal Emission and Reflection Radiometer (ASTER) Global Digital Elevation Model (ASTER GDEM) project is a collaboration between NASA and Japan’s Ministry of Economy, Trade, and Industry (METI). The ASTER GDEM data are collected between 83°N and 83°S. In June 2009, the first version of ASTER GDEM was released from 1.2 million ASTER images. The version was generated from images archived from 2000 to 2008 [33]. In 2011, an improved version was released (ASTER GDEM v2). An additional 260,000 images collected from September 2008 to August 2010, in addition to the initial images were used to produce this improved version [33]. ASTER GDEM version 3 datasets were released on August 5, 2019. Compared to previous versions, ASTER GDEM V3 has fewer elevation voids due to improvements in processing software and the use of a large number of ASTER stereo images (2.3 million scenes). The vertical accuracy of ASTER GDEM v3 in the United States was assessed using 23,000 geodetic ground control points from the U.S. National Geodetic Survey, and the validation yielded a root mean square error (RMSE) of 8.52 meters [34]. The ASTER GDEM v3 data were downloaded from the NASA Earth data portal (https://www.earthdata.nasa.gov/).

### SRTM

The Shuttle Radar Topography Mission (SRTM) 1 arc-second DSM was produced using radar interferometry from space. SRTM mapped land surfaces between 60°N and 56°S latitude, covering about 80% of Earth’s land area. SRTM flew aboard the Space Shuttle mission during an 11-day mission in February 2000, carrying two radar antennas. SRTM provided for the first time a global DEM at 1 arc-second (approximately 30 m) spatial resolution. However, SRTM 1 arc-second global datasets were released later in 2014. The antennas operated in C-band radar (5.6 cm wavelength) and X-band radar (3 cm wavelength) [35]. By capturing radar signals from two slightly different positions, they created stereo radar images of Earth’s surface. This technique is called Interferometric Synthetic Aperture Radar (InSAR). The phase differences between the radar signals from the two antennas were used to calculate the terrain’s elevation with high precision [36]. This produced a dense grid of elevation points. In this study, we used SRTM version 3. The vertical accuracy is expected to be  ± 10 m and ±20 m (RMSE) at 90% confidence for most regions [37]. However, the void-filled version has better accuracy [38]. In addition to providing global coverage, SRTM offers homogeneous quality of the DSM. The SRTM DSM version 3 data were downloaded from the NASA EarthData Portal (https://www.earthdata.nasa.gov/data/).

### NASADEM

NASADEM is an enhanced digital elevation model created by NASA’s Jet Propulsion Laboratory (NASA’s JPL) as an improved version of the original SRTM version 3 [39]. It was officially released in 2020. Unlike earlier SRTM releases, NASADEM reprocessed this raw data using modern algorithms and updated reference datasets. One of the biggest limitations of SRTM was the presence of voids (gaps) caused by radar shadows, steep slopes, and water surfaces. NASADEM fills these gaps using improved interpolation and auxiliary datasets [39]. NASADEM provides a seamless elevation dataset with a 1 arc-second (~30 m) spatial resolution. Studies show NASADEM has better accuracy than the original SRTM, with RMSE typically 5–8 m [12,39].

### Slope, curvature and aspect derivatives

In addition to DSM elevation values, three terrain derivatives were required: slope, curvature, and aspect. Both were calculated from each DEM using ArcGIS’s Slope, Curvature, and Aspect tools, which apply a 3 × 3 moving window to compute the maximum rate of change (slope) and the downslope direction of maximum change (aspect). Slope values were expressed as a percentage. Aspect values were classified into eight cardinal directions (N, NE, E, SE, S, SW, W, NW) for statistical analysis. Using the slope curvature tool, the general (standard) curvature was used in this study. This provided an overall measure of local surface convexity and concavity.

### Reference data

We used the geodetic control points to assess the accuracy of the global DSMs. The data were obtained from the Department of Surveys, Ministry of Lands, Housing and Urban Development, Malawi. The national geodetic control networks were established through Class A surveys, achieving a relative accuracy of 1:12,000 (≈8 mm per 100 m of horizontal distance). The national geodetic control network of Malawi was established progressively between 1948 and 1974. The primary triangulation framework was surveyed mainly during 1948–1953, while secondary and tertiary control networks were developed and densified through 1974 [40]. These monumented points support cadastral and topographic mapping at the national and regional level, representing the highest-precision elevation reference available. We used 2,404 benchmarks with complete three-dimensional coordinates, distributed across all physiographic regions and land cover categories (Supplementary S1 Fig). The national geodetic control points represent bare-earth elevations and therefore provide a high-accuracy terrain reference. When compared with global surface models (ASTER GDEM, SRTM, and NASADEM), observed differences should be interpreted in light of the differing surface representations, particularly in areas with dense vegetation. Originally, the vertical reference system of these benchmarks was tied to the local mean sea level (MSL) defined at the Beira tide gauge (Mozambique). The elevations were subsequently transformed to the Earth Gravitational Model 1996 (EGM96) geoid vertical datum. To harmonise the benchmark elevations with the global geoid-based reference system, a two-stage vertical datum transformation was undertaken. First, the mean offset between the local MSL datum at Beira and the Earth Gravitational Model 2008 (EGM08) was determined to be + 1.01 m [41]. This offset was applied to the original benchmark elevations to convert them from the local MSL reference to EGM2008:


$$\begin{array}{c} {𝐇}_{𝐄𝐆𝐌08}\;=H_{𝐋𝐨𝐜𝐚𝐥}+1.01 \end{array}$$ (1)

Where H_EGM08_ is the target orthometric height aligned with the EGM08 and H_local_: local levelling measurements tied to the mean sea level.

Second, the EGM2008-referenced elevations were transformed to the EGM96 vertical reference system. The difference between EGM2008 and EGM96 was determined at each benchmark location using a geoid calculator (https://earth-info.nga.mil/geograv/dashboard). The resulting point-specific corrections were applied to the EGM08 elevations to obtain benchmark elevations referenced to EGM96.

### Land cover data

We used the 30 m spatial-resolution Global Land Cover Estimation (GLanCE) data downloaded from NASA Earthdata (https://www.earthdata.nasa.gov/data/catalog/lpcloud-glance30-001). The overall accuracy of the product ranges from 75% to 94% across years and continents [42]. Land cover datasets closest to the respective DSM epochs were used to minimise temporal mismatch between vegetation conditions and DSM acquisition dates. A 2001 land cover map was used for SRTM and NASADEM, while a 2010 land cover dataset was used for ASTER GDEM. Although this approach does not fully eliminate temporal inconsistencies, it provides a more representative approximation of land surface conditions at the time of DSM acquisition. The reference national control points fell into the following land-use categories: developed, herbaceous, shrublands, tree cover, and water.

### Data processing and analysis

#### Data clipping and harmonisation.

To ensure comparability across datasets, all DSMs (ASTER, SRTM, and NASADEM) were processed to a common spatial framework. Each DSM was clipped to the Malawi national boundary to remove extraneous data and focus analyses on the study area. The DSMs were reprojected to a uniform spatial reference system, the World Geodetic System 1984 (WGS84), using the Universal Transverse Mercator (UTM) Zone 36S projection in ArcGIS. The nearest neighbour resampling technique was applied to ensure consistent grid alignment across all DSMs. The descriptive statistics before and after grid alignment (Supplementary S1 Table) show small differences in elevation values across all DSMs. The nearest neighbour is preferred for its ability to preserve the original image values and is widely used in DSM analysis [43,44]. In addition, the DSMs were spatially perfectly aligned on the same raster grid. This was verified by ensuring that all DSMs shared the same spatial reference system, pixel resolution, raster extent, and identical grid origin. This ensured that ground control points were spatially matched to the corresponding DSM pixels during elevation extraction.

### Extracting elevation from DSMs

We employed the “Extract Values to Points” tool in ArcGIS to extract elevation values from ASTER, NASADEM, and SRTM DSMs. This assigned the elevation value of the raster cell intersecting the selected 2,404 ground control points. A point-by-point dataset of elevations, derived from DSMs and national geodetic benchmark elevations, was then created and used for direct comparison and error analysis.

### Vertical accuracy assessment

The vertical accuracy assessment was performed by comparing elevations derived from the DSM and elevations from the national geodetic control points. By comparing the two datasets, we determined the mean error (ME), Mean Absolute Error (MAE), Standard Deviation (SD), and Root Mean Square Error (RMSE). The accuracy metrics RMSE, ME, MAE, and SD are widely used to validate the accuracy of DSMs [12,19–21]. Generally, multiple error or accuracy metrics are used to overcome the shortcomings of the others. For instance, RMSE is criticised for its inability to recognise the nature of the error (random, systematic, or blunder) [45]. Additionally, a large number of randomly referenced points are required [46]. To compensate for the shortcomings of RMSE, the use of additional metrics such as ME, MAE, and SD is recommended [47]. These metrics are computed as:


$$\begin{array}{c} {\Delta h}_{i}\;=h_{i,DSM}-h_{i,ref} \end{array}$$ (2)

$$\begin{array}{c} ME\;=\frac{1}{n}\sum_{i=1}^{n}(h_{DSM,i}-h_{Ref,i}) \end{array}$$ (3)

$$\begin{array}{c} MAE\;=\frac{\text{1}}{n}\sum_{i=\text{1}}^{n}{\text{|}h}_{DSM,i}-h_{Ref,i}|\; \end{array}$$ (4)

$$\begin{array}{c} SD\;=\;\frac{\text{1}}{n-\text{1}}\sum_{i=\text{1}}^{n}{\left({\Delta h}_{i}-ME\right)}^{\text{2}} \end{array}$$ (5)

$$\begin{array}{c} RMSE=\sqrt{\frac{1}{n}\sum_{i=1}^{n}{(h_{DSM,i}-h_{ref,i})}^{2}} \end{array}$$ (6)

where the error term (${\Delta h}_{i}$) represents the difference between the DSM-derived elevation ($h_{i,DSM}$) and the measured elevation from ground control points ($h_{i,ref}$) at corresponding locations.

### Sensitivity analysis of neighbourhood effects

We performed a sensitivity analysis using a 3 × 3 focal window to assess the influence of local neighbourhood effects on DSM accuracy. The method evaluates the variability of elevation values extracted from the centre cell compared to those representing the surrounding local neighbourhood. A 3 × 3 focal mean raster was generated using a moving window with a rectangular neighbourhood. Elevation values from the focal mean raster were then extracted at the locations of the geodetic control points and compared with the corresponding centre-pixel values. The ME, MAE, RMSE, and SD were computed for both the centre-pixel and 3 × 3 focal mean datasets. The results are provided in the Supplementary S2 Table.

### Statistical analyses

Two regression approaches were implemented in R to evaluate systematic relationships among DSM elevations, reference elevations, and associated errors for each DSM. We first applied simple linear regression to model the relationship between DSM elevations ($h_{DSM})$ and reference elevations ($h_{ref}$). The simple linear regression is applied as follows:


$$\begin{array}{c} h_{DSM}=\;\beta_{\text{0}}\;+\beta_{\text{1}}h_{ref}+\epsilon \end{array}$$ (7)

The model is used to test if DSM elevations track the reference linearly. When the slope ≈ 1, it indicates unbiased scaling. An ideal DSM should have an intercept ($\beta_{\text{0}}$) close to zero and a slope ($\beta_{\text{1}}$) close to one (indicating unbiased scaling across the elevation range).

The second regression assessed the relationship between elevation errors (∆*h*) and reference elevations ($h_{ref})$ as shown in Equation (8):


$$\begin{array}{c} \Delta h=\;\alpha_{\text{0}}+\;\alpha_{\text{1}}h_{ref}+\;\epsilon \end{array}$$ (8)

The regression model specifically tested for systematic elevation-dependent bias. A nonzero slope ($\propto_{\text{1}}$) would indicate that DSM error varies with elevation. Normally, underestimation at higher elevations or overestimation at lower elevations. The intercept ($\propto_{\text{0}}$) represents the baseline bias across all elevations. As with the first model, diagnostic statistics (*R*^2^, slope and intercept significance, p-values) were reported.

The pairwise statistical tests were conducted to assess whether differences in elevation errors among the DSMs were statistically significant in addition to the regression analyses. Specifically, both parametric and non-parametric approaches were applied. Paired t-tests were used to evaluate whether the mean errors between DSM pairs were significantly different, accounting for the fact that the same 2,404 geodetic control locations were used for all three DSMs. Wilcoxon signed-rank tests were also performed to account for potential non-normality in the error data. The results from both tests were reported using their respective test statistics and p-values to ensure robustness of inference. In addition, Cohen’s d was computed to quantify the magnitude of differences between DSMs, providing a standardised measure of effect size that is independent of sample size. This allowed for interpretation not only of statistical significance but also of practical significance in DSM performance differences across terrain and land cover conditions.

### Random forest analysis of DSM error drivers

A Random Forest (RF) regression analysis was conducted using the randomForest package in R to investigate further the factors influencing DSM accuracy. RF is an ensemble machine-learning algorithm that constructs a large number of decision trees from bootstrap samples of the data and combines their predictions through averaging, thereby improving predictive accuracy and reducing overfitting [48]. The method is particularly effective for modelling complex, non-linear relationships and interactions among variables without requiring strict assumptions about data distributions [49]. Separate RF models were developed for ASTER, SRTM, and NASADEM. Absolute elevation error was used as a response variable, while reference elevation, slope, aspect, curvature, and land cover were predictor variables. The dataset for each DSM was randomly partitioned into 70% training data and 30% testing data. The training subset was used to build the models, and the testing subset was used to evaluate predictive performance and model generalisability. Variable importance was assessed using the percentage increase in mean squared error, a permutation-based metric that quantifies the increase in prediction error when the values of a predictor variable are randomly shuffled while all other variables remain unchanged [48]. Variables producing larger increases in mean squared error were considered more influential in explaining DSM errors. The importance values were subsequently normalised and expressed as percentages to facilitate comparison among predictors and DSMs. Mean importance values across the three DSMs were then calculated and used to rank the relative influence of topographic and environmental factors on DSM accuracy. This approach enabled the identification of the key drivers of DSM error and provided insights into the factors controlling spatial variations in elevation uncertainty.

## Results

### Overall vertical accuracy

The vertical accuracy assessment revealed notable differences among the three DSMs (Table 2). All datasets showed a consistent negative bias, indicating a systematic underestimation of terrain elevation. ASTER showed the largest mean error (−9.56 m), followed by NASADEM (−7.70 m), and SRTM (−6.27 m). In terms of overall accuracy, SRTM performed best, yielding the lowest MAE (13.41 m) and RMSE (19.55 m). This was closely followed by NASADEM (MAE = 13.58 m; RMSE = 19.99 m). ASTER consistently showed the lowest performance across all accuracy metrics (MAE = 15.71 m; RMSE = 22.13 m). The almost similar SD values for SRTM and NASADEM indicate comparable variability in elevation errors. Pairwise comparisons revealed statistically significant differences in elevation values among all three DSMs (Supplementary S3 Table). The 3 × 3 neighbourhood analysis showed negligible differences in accuracy metrics compared to centre-pixel extraction, indicating that sub-pixel variability and edge effects had minimal influence on DSM accuracy (Supplementary S2 Table).

**Table 2:** Summary of vertical accuracy statistics: mean error (ME), mean absolute error (MAE), root mean square error (RMSE), and standard deviation (SD) for three DSMs.

DSM	N	ME (m)	MAE (m)	RMSE (m)	SD (m)
ASTER	2404	−9.56	15.71	22.13	19.97
SRTM	2404	−6.27	13.41	19.55	18.52
NASADEM	2404	−7.70	13.58	19.99	18.46

Regression analyses further supported these findings (Fig 2). For the DSM–Reference elevation regressions, the slopes were close to unity, with values of 0.985, 0.984, and 0.985 for ASTER, SRTM, and NASADEM, respectively. In addition, very high *R*^2^ values (0.9966–0.9971) were recorded. Small but systematic vertical offsets (intercepts) ranged from approximately +4.71 m (ASTER) to +8.66 m (SRTM). Although the regressions produced very high coefficients of determination, these values primarily reflect the large elevation range represented in the dataset and should not be interpreted as evidence of high vertical accuracy. Metrics such as RMSE, MAE, and ME provide a more appropriate assessment of vertical accuracy because they directly quantify elevation differences between DSM and reference elevations.

**Fig 2:**
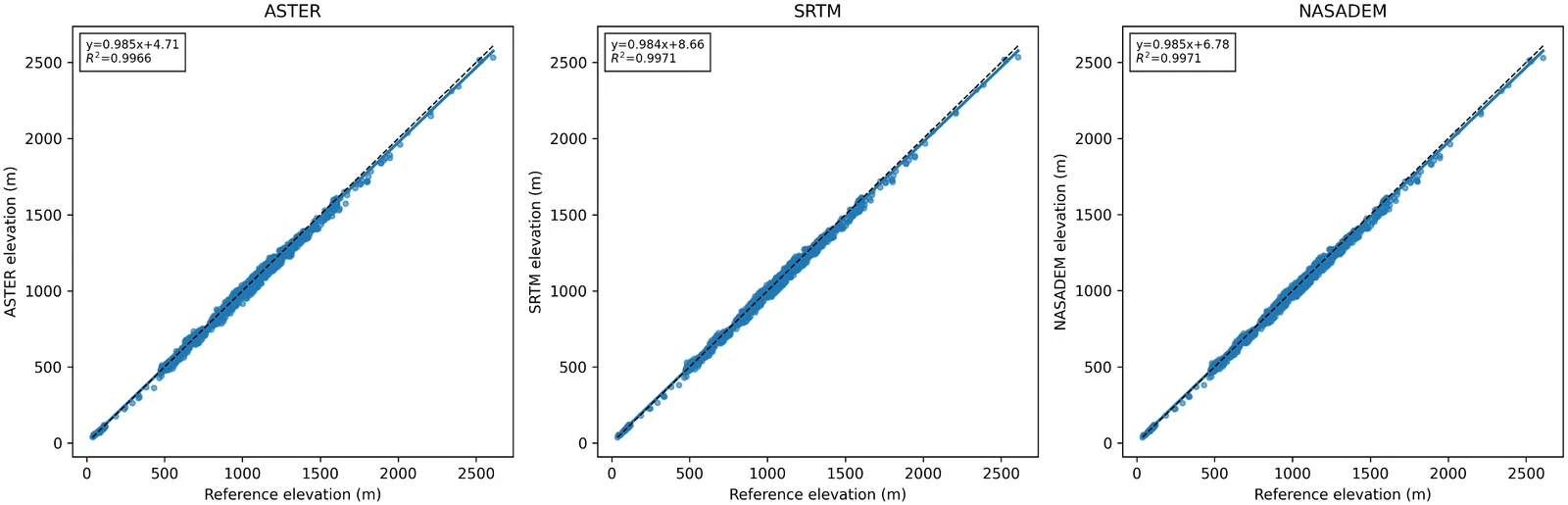
Relationship between DSM-derived elevations and reference elevations for ASTER, SRTM DSM, and NASADEM.

### The effects of topographic variables on the accuracy of DSMs

#### DSM accuracy and aspect.

The influence of terrain aspect on DSM vertical accuracy is summarised in Table 3. Clear directional patterns were observed across all three DSM products, with varying magnitudes and consistency. Overall, the results indicate that the northern and western slopes were consistently associated with underestimation across all DSMs. However, the southern and eastern slopes tended toward smaller errors or slight overestimation. For ASTER, errors are highest in the north-facing slopes (with ME of −17.07 m and RMSE of 27.55 m) and northwest (with ME of −15.22 m and RMSE of 24.72 m). Relatively lower errors are observed in the east (ME = −4.29 m, RMSE = 16.90 m) and south (ME = −1.55 m, RMSE = 17.37 m) for ASTER. SRTM showed overall better accuracy, with positive mean errors in the east (ME = 1.53 m), southeast (ME = 4.94 m), and south (ME = 3.52 m). This suggests slight overestimation in these aspects. However, the errors remained negative in the north and northwest directions, where errors are larger (RMSE up to 23.15 m). NASADEM followed a similar pattern to SRTM but with slightly higher negative bias in northern and western aspects.

**Table 3:** The directional bias in elevation errors across eight aspect classes (N, NE, E, SE, S, SW, W, and NW).

DSM Type	Aspect	n	ME	MAE	RMSE	SD
ASTER	N	328	−17.07	20.28	27.55	21.66
	NE	370	−11.16	15.53	23.18	20.35
	E	248	−4.29	12.03	16.9	16.38
	SE	278	−1.46	13.85	18.7	18.68
	S	220	−1.55	12.62	17.37	17.34
	SW	268	−4.98	14.02	18.96	18.33
	W	297	−14.44	17.66	24.57	19.92
	NW	338	−15.22	17.89	24.72	19.51
SRTM	N	321	−12.18	15.24	22.44	18.88
	NE	337	−9.73	13.96	20.42	17.97
	E	283	1.53	10.24	14.74	14.69
	SE	230	4.94	11.57	16.03	15.28
	S	195	3.52	10.97	14.86	14.48
	SW	227	−2.74	12.07	17.98	17.81
	W	367	−10.81	15.1	21.75	18.9
	NW	373	−14.02	16.62	23.15	18.45
NASADEM	N	333	−14.13	16.15	23.32	18.58
	NE	341	−10.36	13.83	20.42	17.62
	E	278	−0.09	10.3	15.07	15.09
	SE	228	2.6	10.51	15.2	15.01
	S	212	2.17	9.6	13.57	13.42
	SW	240	−3.38	11.63	17.14	16.84
	W	385	−12.63	15.68	22.58	18.74
	NW	369	−14.7	16.91	24.02	19.03

Polar plots of MAE (Fig 3) supported these patterns by visualising error magnitudes around the full azimuth. ASTER showed clear peaks in MAE for northern and western aspects (20.28 m in N and 17.89 m in NW) and minima on east- and south-facing slopes (12.03 m in E and 12.62 m in S). SRTM errors were lowest for east-facing slopes (10.24 m) and highest for northwest-facing slopes (16.62 m), with relatively higher values also in the north (15.24 m) and west (15.10 m). NASADEM exhibited the most uniform MAE distribution, with limited variation across aspects (ranging from 9.60 m (S) to 16.91 m (NW)).

**Fig 3:**
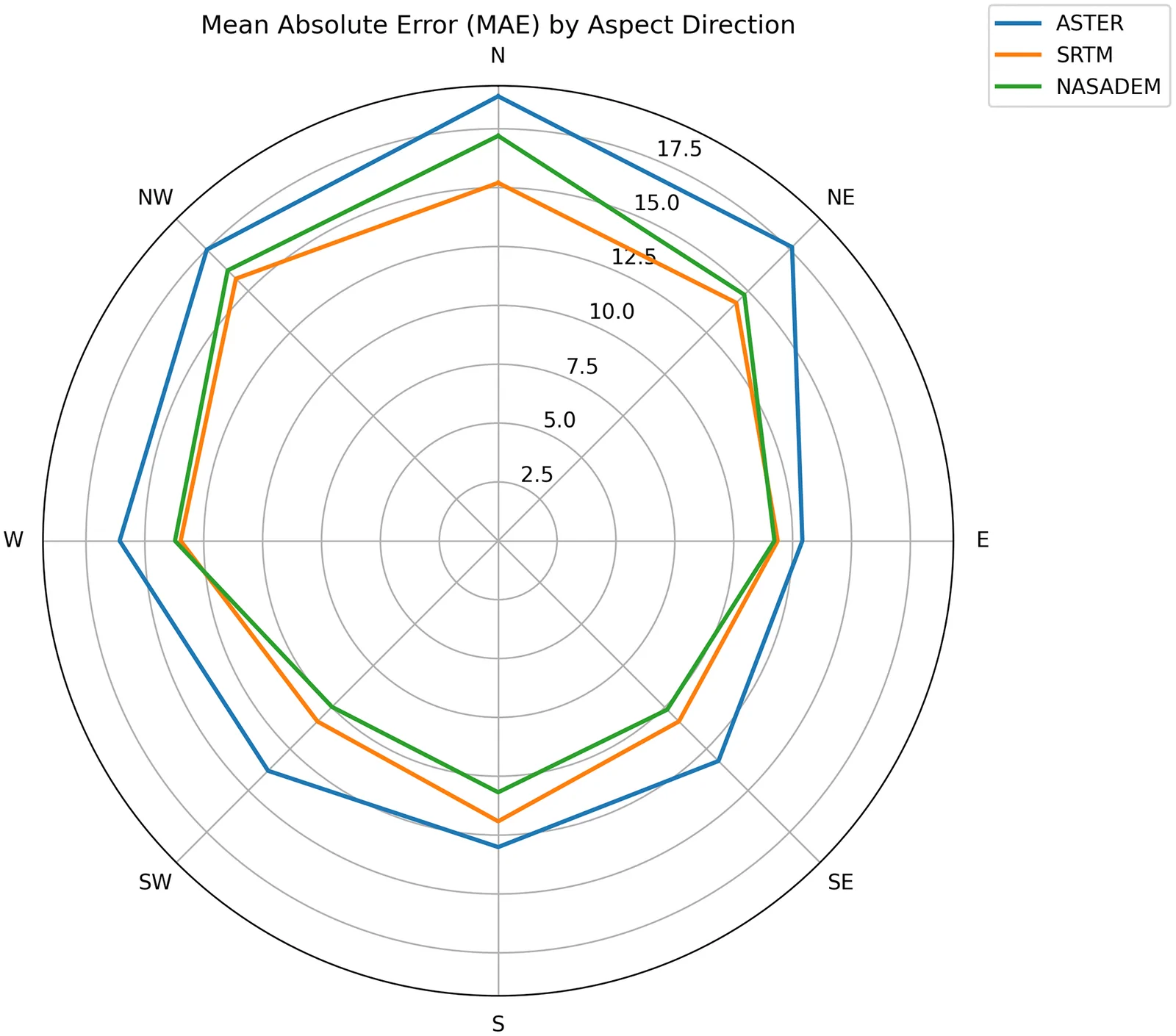
Mean Absolute Error (MAE) by aspect for ASTER, SRTM, and NASADEM.

### DSM accuracy and slope

Table 4 and Fig 4 show how DSM accuracy declines with increasing slope steepness across all three datasets. Both MAE and RMSE remained relatively low in gentle terrains (0–5°), with NASADEM and SRTM performing slightly better than ASTER. However, error magnitudes increased in moderate slopes (5–15°) and steep terrain (15–30°). The errors reached their highest values (RMSE greater than 45 m) in the very steep category (greater than 30°). Comparatively, SRTM and NASADEM show similar performance on gentle to moderate slopes (0–15°), with NASADEM slightly outperforming SRTM in the 0–5° class (MAE = 8.02 m vs 8.18 m). ASTER consistently showed higher errors across all slope classes.

**Table 4:** Vertical accuracy statistics of ASTER, SRTM, and NASADEM across different slope classes in Malawi.

DSM Type	Slope	n	ME (m)	MAE (m)	RMSE (m)	SD (m)
ASTER	0–5	728	−8.37	12.21	17.24	15.00
	5–15	1202	−7.83	13.95	19.29	17.63
	15–30	401	−12.61	22.73	29.79	27.01
	>30	73	−33.09	41.04	46.57	34.53
SRTM	0–5	1018	−3.36	8.18	12.53	12.06
	5–15	1087	−5.28	14.00	19.00	18.27
	15–30	242	−15.51	26.13	31.98	28.19
	>30	57	−38.05	41.50	45.79	25.47
NASADEM	0–5	904	−4.59	8.02	12.74	12.05
	5–15	1217	−6.83	13.78	19.02	17.78
	15–30	224	−17.28	27.09	32.69	27.74
	>30	59	−36.82	43.38	47.73	32.43

**Fig 4:**
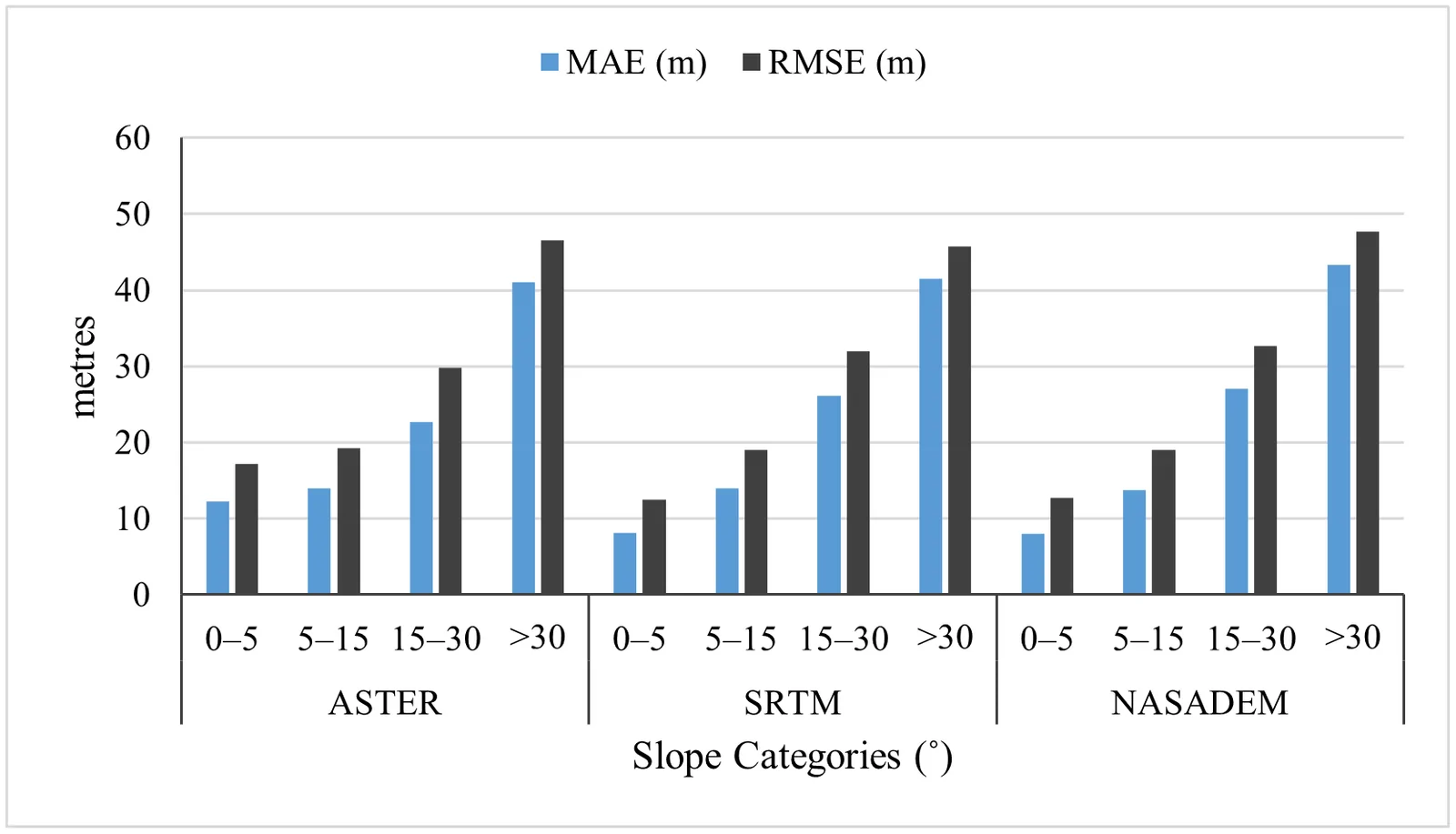
Mean Absolute Error (MAE) and Root Mean Square Error (RMSE) of ASTER, SRTM, and NASADEM across slope categories in Malawi.

### DSM accuracy and curvature

The curvature-based analysis indicates that DSM accuracy varies systematically with terrain form (Table 5). All three datasets showed negative mean errors and increasing error magnitude from concave to convex surfaces. The lowest errors were consistently observed in strongly concave and moderately concave areas (e.g., MAE ≈ 7–13 m; RMSE ≈ 12–19 m). Nearly flat terrain showed intermediate accuracy. In contrast, accuracy deteriorated substantially in convex terrain (moderately and strongly convex classes), where MAE increased to about 25–41 m, and RMSE reached up to 47 m. NASADEM and SRTM generally performed similarly and better than ASTER in concave and flat areas. ASTER showed consistently higher errors across all curvature classes and with the highest errors in convex landforms.

**Table 5:** Vertical accuracy statistics of ASTER, SRTM, and NASADEM across different curvature classes in Malawi.

DSM Type	Curvature	n	ME (m)	MAE (m)	RMSE (m)	SD (m)
ASTER	Strongly Concave (<−1.0)	12	−6.21	13.44	17.86	16.77
	Moderately Concave (−1.0 to −0.3)	198	−7.54	13.21	18.91	17.46
	Nearly Flat/Planar (−0.3 to 0.3)	1766	−8.41	13.98	19.24	17.53
	Moderately Convex (0.3 to 1.0)	401	−15.78	25.06	32.91	29.92
	Strongly Convex (> 1.0)	27	−30.62	39.41	45.18	33.78
SRTM	Strongly Concave (<−1.0)	9	−2.81	7.98	12.42	11.61
	Moderately Concave (−1.0 to −0.3)	220	−3.73	11.12	15.85	15.40
	Nearly Flat/Planar (−0.3 to 0.3)	1708	−5.41	13.02	18.07	17.27
	Moderately Convex (0.3 to 1.0)	412	−18.63	28.40	34.96	31.20
	Strongly Convex (> 1.0)	24	−34.57	39.28	44.62	29.44
NASADEM	Strongly Concave (<−1.0)	10	−3.16	7.64	11.93	11.11
	Moderately Concave (−1.0 to −0.3)	215	−4.92	11.67	16.32	15.60
	Nearly Flat/Planar (−0.3 to 0.3)	1741	−6.63	13.36	18.21	17.01
	Moderately Convex (0.3 to 1.0)	406	−19.87	29.55	35.84	31.58
	Strongly Convex (> 1.0)	25	−33.88	41.03	47.19	32.84

### DSM accuracy and land cover

Table 6 and Fig 5 summarise the vertical accuracy of ASTER, SRTM, and NASADEM across different land cover types in Malawi. In all land cover classes, all DSMs consistently underestimated ground elevations relative to the reference data. The magnitude of these errors varied substantially across land cover types, with developed areas showing the highest accuracy and tree-covered areas the lowest. ASTER, SRTM, and NASADEM produced the highest accuracies (RMSE) of 15.82 m, 13.22 m, and 13.40 m, respectively, in developed areas. The lowest accuracy (RMSE) occurred in tree-covered areas, where RMSEs of 32.26 m (ASTER), 30.05 m (SRTM), and 30.79 m (NASADEM) were observed. SRTM generally achieved the lowest RMSE values across most land cover classes among the three DSMs, followed closely by NASADEM. ASTER consistently exhibited the highest errors, particularly in vegetated environments. Water was removed because of the low number of samples.

**Table 6:** Vertical accuracy statistics of ASTER, SRTM, and NASADEM across different land cover classes in Malawi.

DSM	Land cover	n	ME	MAE	RMSE	SD
ASTER	Developed	802	−5.20	11.81	15.82	14.95
	Herbaceous	1015	−12.08	16.20	22.83	19.38
	Shrublands	390	−9.40	18.17	25.55	23.78
	Tree Cover	184	−14.87	25.18	32.26	28.70
SRTM	Developed	766	−1.62	9.50	13.22	13.13
	Herbaceous	1030	−8.66	13.68	20.01	18.05
	Shrublands	413	−6.86	15.58	22.44	21.40
	Tree Cover	180	−10.81	23.74	30.05	28.12
NASADEM	Developed	766	−3.07	9.42	13.40	13.05
	Herbaceous	1030	−10.01	13.99	20.53	17.94
	Shrublands	413	−8.25	15.64	22.83	21.32
	Tree Cover	180	−12.45	24.27	30.79	28.24

**Fig 5:**
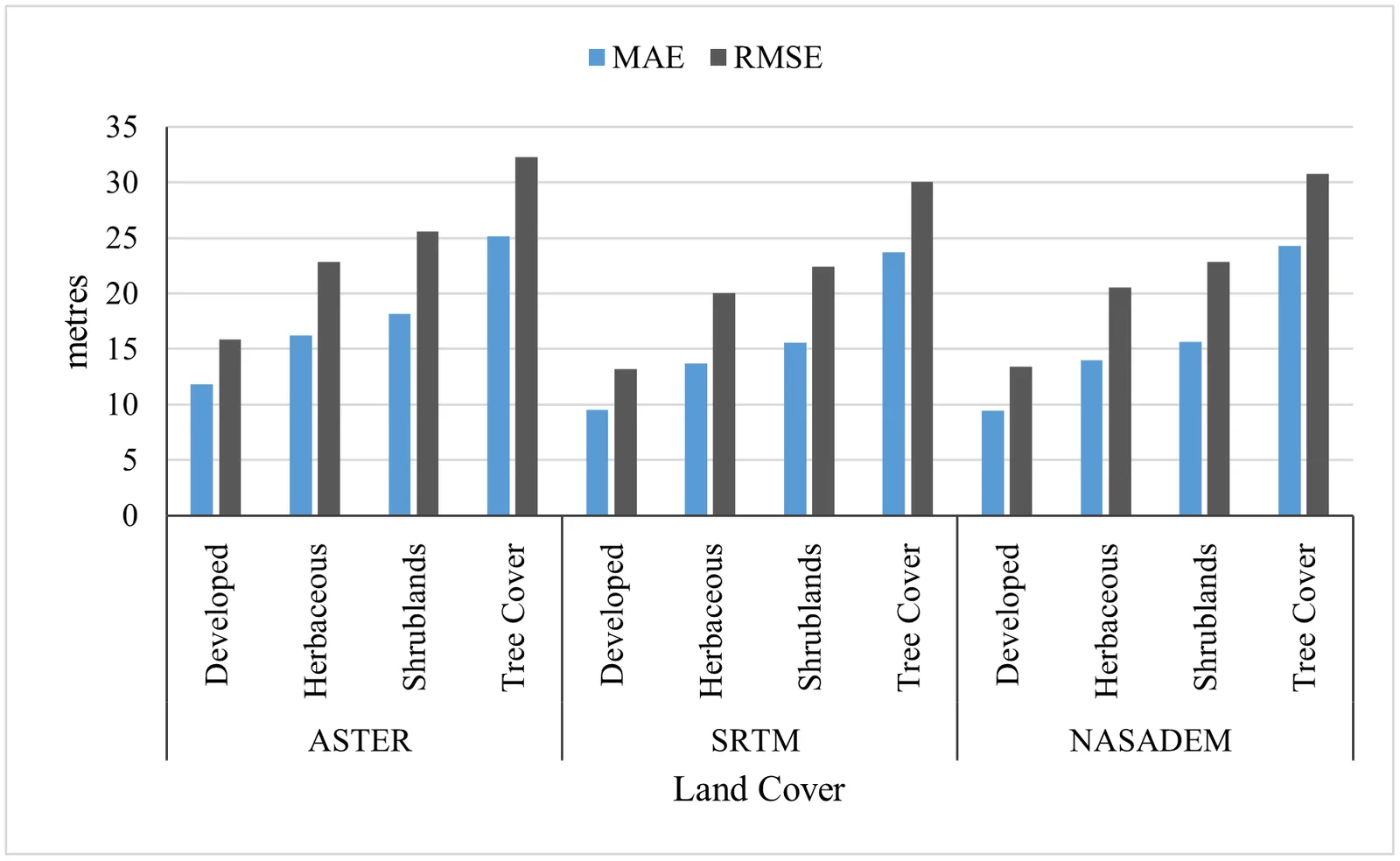
Comparison of vertical accuracy of ASTER, SRTM, and NASA Digital Surface Models across different land cover types.

### Influence of topographic region on DSM accuracy

DSM accuracy varied strongly across Malawi’s topographic regions (Table 7 and Fig 6). The lowest errors were recorded in the low-lying Lower Shire Valley and Lake Shore and Middle & Upper Shire regions. The largest discrepancies were found in mid-elevation and highland areas. NASADEM achieved the best overall accuracy, particularly in the Lower Shire Valley (MAE = 2.36 m, RMSE = 3.25 m). However, errors increased substantially in the higher terrain regions (MAE = 27.26 m) with an accuracy (RMSE) of 35.03 m. SRTM showed comparable performance to NASADEM in the lowlands (MAE = 3.52 m, RMSE = 4.30 m) but also deteriorated in the highlands (MAE = 25.92 m, RMSE = 33.62 m). ASTER consistently showed the largest errors in all the topographic regions relative to the other two DSMs. For all three DSMs, elevation errors generally increased with increasing terrain elevation.

**Table 7:** Vertical accuracy statistics of ASTER, SRTM, and NASADEM across different topographic regions of Malawi.

DSM Type	Topographic Region	N	ME	MAE	RMSE	SD
ASTER	Lower Shire Valley (<200 m)	74	−3.70	5.65	6.91	5.87
	Lake Shore and Middle & Upper Shire (200 m-760 m)	643	−5.88	11.15	16.44	15.37
	Mid-Elevation (760 m-1300 m)	1477	−8.71	16.21	21.98	20.19
	Highlands (>1300 m)	210	−28.85	29.70	36.94	23.13
SRTM	Lower Shire Valley (<200 m)	74	2.62	3.52	4.30	3.44
	Lake Shore and Middle & Upper Shire (200 m-760 m)	643	−3.07	8.74	13.53	13.19
	Mid-Elevation (760 m-1300 m)	1477	−5.49	14.15	19.52	18.74
	Highlands (>1300 m)	210	−24.73	25.92	33.62	22.83
NASADEM	Lower Shire Valley (<200 m)	74	0.62	2.36	3.25	3.21
	Lake Shore and Middle & Upper Shire (200 m-760 m)	643	−4.85	8.65	13.74	12.87
	Mid-Elevation (760 m-1300 m)	1477	−6.68	14.34	19.83	18.68
	Highlands (>1300 m)	210	−26.50	27.26	35.03	22.97

**Fig 6:**
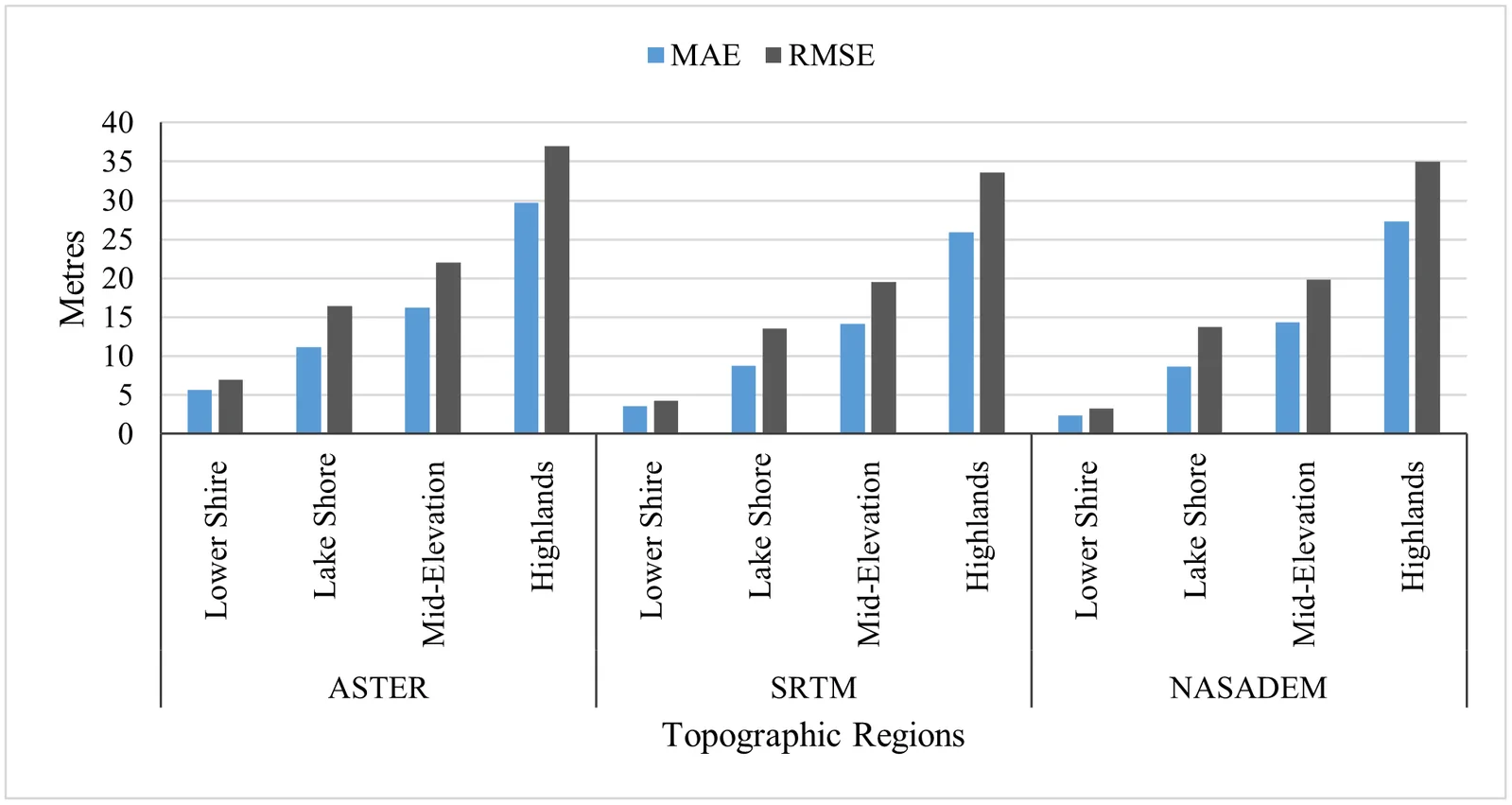
Comparison of vertical accuracy of ASTER, SRTM, and NASADEM across different topographic regions in Malawi.

### Relative importance of factors influencing DSM errors

The Random Forest variable importance analysis shows a consistent hierarchy of factors controlling DSM errors across ASTER, SRTM, and NASADEM datasets (Table 8). Aspect emerged as the most influential predictor, with the highest mean contribution (38.80%). This effect was particularly higher in SRTM and NASADEM, where 42.11% and 41.26% of DSM errors can be explained by aspect, respectively. Elevation was the second most important factor, with a mean contribution of 36.23%. Particularly for ASTER, elevation contributed 43.20%. Slope demonstrated moderate importance with a mean contribution of 18.11%. Land cover and curvature emerged as relatively minor drivers, with mean contributions of 4.94% and 1.93%, respectively.

**Table 8:** Relative importance of factors influencing DSM errors.

Variable	ASTER (%)	SRTM (%)	NASADEM (%)	Mean (%)	Rank
Aspect	33.02	42.11	41.26	38.80	1
Elevation	43.20	31.39	34.08	36.23	2
Slope	16.60	19.61	18.12	18.11	3
Land cover	5.10	5.05	4.67	4.94	4
Curvature	2.08	1.84	1.86	1.93	5

## Discussion

### Overall accuracy of DSMs

This study assessed the vertical accuracy of three widely used global DSMs (ASTER GDEM, SRTM, and NASADEM) across diverse landscapes in Malawi using national geodetic benchmarks. Our results show that SRTM has the best vertical accuracy, followed by NASADEM. SRTM yielded the lowest national RMSE (19.55 m), followed closely by NASADEM (19.99 m). Although pairwise tests indicated statistically significant differences, the magnitude of the national-scale RMSE difference between SRTM and NASADEM was small (0.44 m). NASADEM, a successor to the SRTM, did not display improved accuracy at the national level. However, in the low-lying regions of Malawi, NASADEM outperformed SRTM. Improvements in NASADEM are often region-specific and may not always translate into substantial gains in vertical accuracy [50]. While several studies have shown NASADEM to outperform SRTM in many regions [12,19,39], the results in different topographic regions in Malawi indicate that such improvements are not universal. The study also revealed systematic underestimation and larger errors in ASTER-derived elevations in all topographic regions in Malawi. The errors increased significantly in steep and elevated terrain. Cloud cover and the data artefacts are some of the factors that contribute to the relatively low accuracy in ASTER [51,52]. This is typical in areas with fewer available stereo images used for processing. The variations in the accuracy of the studied DSMs confirm that their performance and reliability differ, despite all offering valuable elevation information.

The results of this study both agree with and differ from previous assessments of global elevation products. This highlights the influence of geographic, topographic, and environmental context on DSM performance. For instance, NASADEM, the successor of SRTM, only produced marginal accuracy improvement with RMSEs of 13.07 m, 10.00 m, and 6.59 m in New Zealand, China, and Estonia, respectively [21]. A DEM validation study in Malaysia found NASADEM slightly more accurate overall than SRTM, but the improvement was minor [53]. Slightly better performance for NASADEM than SRTM was observed in Iran, further suggesting that the relative performance of these closely related products may vary according to geographic and environmental context [54]. In Mexico, NASADEM was validated with an RMSE of 5.2 m [47]. The overall RMSE of 19.55 m of SRTM observed in this study is comparable to values reported in other studies conducted in topographically complex environments. For example, Sharma et al. [55] reported an RMSE of 27.58 ± 4.22 m for SRTM in eastern India. Mukul et al. [56] found RMSE values of 23.53 m in the Indian Himalaya. On the contrary, the RMSE of SRTM we have reported is higher than the 10.39 m reported in southwestern China [14] and 2.88 m in India [57]. In addition, the relatively high overall RMSE of ASTER GDEM observed in this study (22.13 m) is consistent with previous studies. For example, LiDAR-based validation in Greenland reported ASTER vertical errors ranging from 15 m to 65 m [58]. The relatively low performance of ASTER GDEM observed in this study also agrees with the findings of Yap et al. [59], who reported RMSE values of 18.87 m for ASTER GDEM compared with 13.25 m for SRTM in Cameroon. The RMSE value of 18.56 m for ASTER was reported in southwestern China [14] and is higher than 8.52 m reported over the conterminous United States [34]. Li et al. [60] suggested that the accuracy of ASTER GDEM can be significantly improved through correction with ICESat-2 altimetry data. Their results suggest that the relatively lower accuracy of ASTER observed in this study is partly attributable to limitations of the original stereo-derived product, particularly in complex terrain and vegetation-covered landscapes.

### The effects of topographic and land cover variables on DSM’s accuracy

The random forest analysis indicates that aspect was the most influential factor affecting DSM accuracy, contributing an average of 38.80% across ASTER, SRTM, and NASADEM. The study established that DSM errors tended to be slightly larger on north- and west-facing slopes. ASTER was particularly sensitive to aspect effects, with RMSE values exceeding 27 m on north-facing slopes compared with approximately 17 m on southern slopes. Similar patterns were observed for SRTM and NASADEM, although the magnitude of the differences was smaller. This may reflect directional biases in sensor geometry and illumination [14]. On the contrary, Szabó et al. [61] observed that SRTM and ASTER had the largest errors occurring on slopes facing the southern and eastern directions. The tendency in ASTER was attributed to the method used in surface generation, which, given the high number of images per database part, results in local differences. In addition, this may reflect directional biases in sensor geometry and illumination [14]. These findings align with results from other studies that highlight vegetation cover and illumination geometry as important factors affecting DSM accuracy [14,47,62,63].

Terrain height is one of the factors that affect DSM accuracy. The study has shown that elevation had an overall contribution of 36.23% to DSM errors and ranked second. It was the dominant predictor for ASTER (43.20%). This suggests that absolute terrain height strongly influences DSM accuracy, particularly for optical stereo-derived DSMs like ASTER GDEM. Higher elevations are commonly associated with greater terrain ruggedness, steeper slopes, increased relief displacement, and more difficult image matching. Radar-derived DSMs are also affected by elevation because terrain height alters incidence angles and backscatter geometry. Differences in DSM errors and accuracies were observed across the four topographic regions of Malawi. The low-lying topographic region (elevation <200 m), the lower Shire Valley, performed best across all three DSMs. NASADEM outperformed other DSMs with MAE and RMSE values of 2.36 m and 3.25 m, respectively, in this region. Despite the highest performance of NASADEM in this region, it displayed no improvement over SRTM at the national level. On the contrary, the lowest performance for all the DSMs was recorded in the highland regions (elevation >1300 m). All the DSMs underestimated elevations by an average of more than 25 m. ASTER, SRTM, and NASADEM registered their highest RMSEs of 36.94 m, 33.62 m, and 35.03 m in the highlands, respectively. Several global DSM validation studies have similarly shown that elevation is a major determinant of DSM accuracy. For example, a global DSM assessment demonstrated that accuracy varies considerably with topographic setting, particularly in mountainous areas where elevation and terrain complexity increase [21]. Similarly, the highest RMSE was observed in the mountainous region in South Africa [63]. On the contrary, Yao et al. [14] noted that the vertical accuracy of ASTER and SRTM did not vary with elevation in the Tibetan Plateau Mountain Ranges.

Slope was the third most important variable according to the RF results and contributed an average of 18.11% across all three DSMs. Generally, steeper slopes increase vertical uncertainty because even small horizontal displacement errors translate into larger vertical differences. The slightly higher importance of slope for SRTM (19.61%) compared with ASTER (16.60%) and NASADEM (18.12%) indicates that radar-derived DSMs remain sensitive to terrain steepness despite the refinements introduced in NASADEM. In slopes between 0° and 5°, the RMSEs were 12.53 m, 12 m, and 17.24 m for SRTM, NASADEM, and ASTER, respectively. However, the RMSEs exceeded 45 m in slopes more than 30° for all three DSMs. These trends are consistent with the physical principles of DSM acquisition of radar interferometry used to capture SRTM/NASADEM [64]. Radar interferometry suffers from layover and shadow in steep terrain [64]. On the other hand, ASTER’s optical stereo method is prone to image matching errors under complex illumination and topographic shadowing [51,52]. Such elevation-related trends have been widely reported in Estonia, Norway, New Zealand, and China [21] and Thailand [35]. Toutin [65] reported an almost linear relationship between radar DEM errors and terrain slope, with the largest errors occurring on steep slopes. The influence of terrain on DSM accuracy observed in this study is also consistent with the findings of Wang et al. [66], who demonstrated that increasing slope and terrain relief resulted in larger elevation errors across multiple open-access DEMs. Their work further demonstrated the need to consider local terrain conditions when selecting elevation datasets for geospatial applications.

In contrast, land cover contributed only 4.94%, indicating that vegetation and surface cover exerted relatively little influence compared with terrain variables. Although vegetation height and canopy structure can influence both radar penetration and optical image correlation, their effects appear secondary in the present study because topographic controls dominate the spatial distribution of DSM errors. The relatively consistent importance across ASTER, SRTM, and NASADEM further suggests that the influence of land cover is similar regardless of DSM generation method. Despite the low contribution of land cover to the DSM errors, variations in error distribution across different land cover types were visible. The highest accuracies were observed in the developed land cover category, with RMSEs of 15.85 m (ASTER), 13.22 m (SRTM), and 13.40 m (NASADEM). However, accuracies substantially declined in the tree-covered land cover, with RMSEs of 32.26 m (ASTER), 30.05 m (SRTM), and 30.79 m (NASADEM). The higher errors in landscapes covered with trees reflect the radar signal penetration capability in SRTM/NASADEM [23]. Radar techniques tend to capture canopy surfaces rather than bare ground [35,36]. It is important to recognise that the reference elevations used in this study represent bare-earth terrain, while the assessed datasets are more appropriately described as DSM. This fundamental difference introduces a systematic component to the observed elevation discrepancies in vegetated and complex landscapes where surface elevations exceed true ground levels. Consequently, part of the measured error reflects differences between surface and terrain representations rather than purely sensor inaccuracy. Despite this limitation, the analysis was designed to explore the combined influence of terrain morphology and surface conditions on elevation differences, rather than to isolate bare-earth accuracy alone. In addition, the relatively low contribution of land cover in the Random Forest models suggests that, within the study area, large-scale topographic variables (e.g., aspect, elevation, and slope) dominate over vegetation effects. Therefore, the results should be interpreted as representing elevation discrepancies driven by both terrain characteristics and surface structure, rather than strictly as errors relative to a true bare-earth DEM.

Curvature was the least influential predictor, accounting for only 1.93% of the total importance. DSM resolution becomes coarser, fine-scale terrain features such as ridges, gullies, and small depressions are progressively smoothed, resulting in a reduced range of curvature values and a diminished ability to capture local terrain variability [67,68]. Larger-scale terrain attributes explain substantially more variation in elevation errors than local morphometric parameters such as curvature. Although many of the errors cannot be explained by curvature, they varied across different curvature types. The errors were low in nearly/planar areas and increased in the strongly concave and convex curvatures in all DSMs. Strongly convex curvature displayed the lowest accuracy. Similar trends were also observed where NASADEM performed very well in flat areas and in steep, rugged mountains, and errors rose sharply [21]. Crippen et al. [50] also noted that NASADEM tended to slightly overestimate ridges and underestimate valleys. However, estimates for the strongly concave and strongly convex curvature classes should be interpreted cautiously, as the limited sample sizes may reduce the reliability and robustness of the results.

The principal contribution of this study is not simply the identification of the DSM with the lowest national RMSE, but the demonstration that vertical accuracy is strongly conditioned by landscape context. Although SRTM achieved the lowest national RMSE (19.55 m), the difference between SRTM and NASADEM at the national scale was relatively small (0.44 m). In contrast, much larger variations in accuracy were observed across Malawi’s physiographic regions, with RMSE values ranging from less than 5 m in the Lower Shire Valley to more than 35 m in the Highlands. This finding challenges the common practice of treating nationally averaged accuracy statistics as representative of DSM performance throughout an entire country. Instead, the results indicate that DSM suitability should be viewed as a spatially varying property controlled by terrain configuration, elevation, slope, and surface characteristics among other factors. Consequently, the selection of a DSM should be guided not only by overall accuracy metrics but also by the environmental conditions in which the dataset will be applied.

## Conclusion

This study provides a national-scale assessment of the vertical performance of ASTER GDEM, SRTM and NASADEM across Malawi using a common set of 2,404 national geodetic control points. The principal scientific contribution is the demonstration that the accuracy of global DSMs is strongly terrain-dependent and that national-scale accuracy statistics alone can conceal substantial spatial variation in elevation uncertainty. SRTM (RMSE = 19.55 m) and NASADEM (RMSE = 19.99 m) showed comparable performance, whereas ASTER produced a higher RMSE of 22.13 m at the national scale.

The terrain-stratified analysis revealed much larger differences than those observed at the national scale. NASADEM performed particularly well in the Lower Shire Valley with an RMSE of 3.25 m. However, the RMSE increased to 35.03 m in the Highlands. Similarly, SRTM increased from 4.30 m in the Lower Shire Valley to 33.62 m in the Highlands, while ASTER increased from 6.91 m to 36.94 m. Additionally, accuracy also generally decreased with increasing elevation and slope, with the largest errors occurring in high-relief terrain. The Random Forest analysis further indicated that aspect, elevation and slope were the most influential predictors of DSM error. Tree-covered areas exhibited substantially greater errors than other land cover types.

These findings provide scientific evidence that the performance of global DSMs should be understood as spatially variable rather than uniform across a country. The results therefore extend previous global and regional DSM validation studies by demonstrating that the practical difference between products at the national scale may be considerably smaller than the variation in accuracy caused by terrain and surface conditions within the same country.

The findings also have practical value for geospatial applications in Malawi. SRTM and NASADEM may provide useful elevation information for applications in lowland and moderately sloping environments. The use of these products in highland, steep and densely vegetated areas should be accompanied by greater caution. In addition, the products should undergo local validation or bias correction where possible. Applications that are sensitive to vertical elevation error (e.g., hydrological modelling, flood and erosion assessment, land-use planning, infrastructure planning and terrain analysis) may be particularly affected by the spatial variation identified in this study. Therefore, users should consider locally validated or higher-resolution elevation datasets where higher accuracy is required.

### Limitations and future work

Despite the use of highly accurate national geodetic benchmarks, some uncertainties remain. Firstly, the national geodetic control points represent bare-earth elevations, whereas the evaluated datasets are DSMs that may capture vegetation canopies and other above-ground features. Consequently, elevation differences observed in vegetated and built-up areas should be interpreted not only as DSM errors but also as differences arising from the contrasting surface representations. Secondly, the spatial distribution of reference data may not fully capture all combinations of terrain and land cover. We acknowledge that the distribution of control points reflects the design of the national geodetic network rather than a statistically uniform sampling scheme. Hence, some land cover classes (e.g., water) and topographic categories (e.g., strongly convex and strongly concave curvature) had inadequate sample sizes. The limited sample sizes may reduce the reliability and robustness of the results.

Another limitation is the potential temporal mismatch between the acquisition dates of the global DSMs and the land cover conditions represented by the reference data. To minimise this effect, land cover datasets closest to the respective DSM acquisition periods were used. However, some temporal discrepancies remain. This is particularly the case in areas that have undergone land cover change or vegetation growth since satellite observations began. It is worthy to note that some of the elevation residuals observed in vegetated areas may reflect real changes in land cover over time rather than solely the vertical accuracy of the DSMs. Therefore, the results for land cover classes, particularly tree cover, should be interpreted with this limitation in mind. Future studies should incorporate time-specific land cover datasets to better account for vegetation dynamics. In addition, slope and aspect were derived from the same DSMs used for the accuracy assessment, as no independent high-resolution Digital Terrain Model was available. This introduces a degree of circularity. Additionally, the study did not assess the ASTER GDEM NUM layer (scene count per pixel). This means that the potential spatial variability in scene density and its effect on local accuracy was not explicitly quantified. Therefore, future work should explicitly incorporate NUM-layer analysis to further assess the relationship between scene density and elevation accuracy. Lastly, this study focused on three widely used DSMs at 30 m resolution. The performance of newer high-resolution DEMs (e.g., TanDEM-X, ALOS AW3D30, Copernicus DEM) should therefore be evaluated to benchmark global DEM/DSM accuracy in Malawi and to refine national geospatial datasets.

## Supporting information

S1 Table Summary statistics of the ASTER, SRTM, and NASADEM digital surface models (DSMs) before and after resampling. The table reports the minimum, maximum, mean, and standard deviation of elevation values (m) for each dataset, demonstrating that resampling preserved the overall elevation distributions with only negligible changes in summary statistics.
(XLSX)

S2 Table Comparison of elevation accuracy metrics for ASTER GDEM, SRTM, and NASADEM using centre-pixel extraction and a 3 × 3 focal mean extraction method. The table presents mean error (ME), mean absolute error (MAE), root mean square error (RMSE), and standard deviation (SD), showing that the two extraction methods produced nearly identical accuracy estimates across all three DSMs.
(XLSX)

S3 Table Statistical comparison of elevation errors between ASTER GDEM, SRTM, and NASADEM based on paired t-tests, Wilcoxon signed-rank tests, and paired Cohen’s d effect sizes. The results indicate statistically significant differences in elevation errors among all DSM pairs, with effect sizes ranging from small to large.
(XLSX)

S1 Fig Spatial distribution of the national geodetic control points used as reference elevation data across Malawi. The figure illustrates the geographic coverage of the control points, demonstrating their distribution across the country’s diverse topographic regions and providing the reference dataset for assessing the vertical accuracy of the ASTER GDEM, SRTM, and NASADEM digital surface models.
(JPG)
