# Supplementary material for: Vertical accuracy of ASTER, SRTM, and NASADEM digital surface models across Malawi’s diverse topography
Source: PLoS One. 2026 Oct 29;21(10):e0361397. doi: 10.1371/journal.pone.0361397 (PMC13718972; doi:10.1371/journal.pone.0361397)
Supplement: Supplementary file 2 [file pone.0361397.s007.docx]

**RESPONSES TO REVIEWER’S COMMENTS**

| 1. Please ensure that your manuscript meets PLOS ONE's style requirements, including those for file naming. The PLOS ONE style templates can be found at  https://journals.plos.org/plosone/s/file?id=wjVg/PLOSOne_formatting_sample_main_body.pdf and  [https://journals.plos.org/plosone/s/file?id=ba62/PLOSOne_formatting_sample_title_authors_affiliations.pdf](https://track.editorialmanager.com/CL0/https:%2F%2Fjournals.plos.org%2Fplosone%2Fs%2Ffile%3Fid=ba62%2FPLOSOne_formatting_sample_title_authors_affiliations.pdf/1/010f019e0049866d-09f494df-69e0-4f46-945e-e2560c597a73-000000/tnTTdGDEsEyw2HKOl0OPvLEVpou220-Y3SI3sBIWLac=258) | Thank you for bringing this to our attention. We have carefully revised the manuscript to ensure that it complies with the PLOS ONE style requirements, including formatting, file naming, and manuscript structure. We believe that now the manuscript fully adheres to the guidelines. |
| --- | --- |
| 2. Please note that PLOS One has specific guidelines on code sharing for submissions in which author-generated code underpins the findings in the manuscript. In these cases, we expect all author-generated code to be made available without restrictions upon publication of the work. Please review our guidelines at https://journals.plos.org/plosone/s/materials-and-software-sharing#loc-sharing-code and ensure that your code is shared in a way that follows best practice and facilitates reproducibility and reuse. | Thank you for your comment. We have made all author-generated scripts used for the statistical analyses accessible through: [10.5281/zenodo.21161092](https://doi.org/10.5281/zenodo.21161092) |
| 3. Please note that your Data Availability Statement is currently missing the DOI/accession number of each dataset OR a direct link to access each database. If your manuscript is accepted for publication, you will be asked to provide these details on a very short timeline. We therefore suggest that you provide this information now, though we will not hold up the peer review process if you are unable. | Thank you for your comment. We have updated the Data Availability Statement to include the DOI for the publicly accessible dataset. The data supporting the findings of this study are available through Zenodo at <https://doi.org/10.5281/zenodo.21101053>. |
| REVIEWER 1 |  |
| a) ensuring that the distinction of DSM vs DEM and the interpretation of the results in such a distinction (.i.e. control points are bare earth elevations, SRTM is not a bare earth elevation). The issue is mentioned by the authors themselves in line 431 – 433, where it is pointed out that SRTM is a DSM (tree canopy is “seen”) but this issues is not dealt with during the analysis. | We thank the reviewer for this valuable comment. We have revised the manuscript to clearly distinguish between the bare-earth reference data and the DSM characteristics throughout the Methods and Discussion. In addition, we now consistently refer to the evaluated datasets as DSMs rather than DEMs. We have also clarified in our discussion that low accuracy in tree land cover may be an inherent nature of DSMs. |
| b) the presentation of all the data and techniques. In its present state I could not repeat the study as I do not have any information on the Malawian geodectic control points (or know who to contact to get them should they be considered sensitive); nor do I know how the DSMs were prepared prior to analysis (i.e. how they were resampled, how the were reprojected). | We thank the reviewer for this helpful comment. The manuscript has been revised to improve the description of the datasets and preprocessing workflow. We have expanded the Reference Data section to describe the source and vertical data conversion (Lines 196-202). We have also provided additional details on the DSM preprocessing (e.g. the use of nearest neighbour resampling during reprojection) (Lines 262-270). The data can be accessed from the following link: [10.5281/zenodo.21101053](https://doi.org/10.5281/zenodo.21101053). . |
| Authors need to differentiate between digital surface model and digital terrain model as it has implications for the interpretation of their results. SRTM and NASADEM are both derived from RADAR interferometry, and do not represent a bare earth surface model (i.e. a “true” DEM); similarly ASTER GDEM is based on stereo scence. Consequently they are DSM, i.e. dense canopy and buildings contribute to the height values of individual cells. See for example, Jacobsen and Passini (2010) and Simard et al. (2024) | We agree that a clear distinction between DTMs and DSMs is essential for correct interpretation of results. In the revised manuscript, we explicitly classify SRTM, NASADEM, and ASTER GDEM as satellite-derived DSMs.We have revised the terminology throughout. We have also updated the interpretation of elevation differences, most especially in vegetated areas (564-576, 622-6269). |
| Supplementary table of points assess I need.  e.g. Point Name, Lat, Long, Control Point Elevation, DEM 1 Elevation, DEM 2 Elevation, DEM 3 Elevation  These points should also be shown a on Supplementary map.  Reason is that not indication of spread of points is given, there may be a bias in spatial distribution of points that may influence the result. Such point geographic distribution needs to be addressed in text. For example, where are the “Water” land cover type (Figure 6) points located ?  Similar for Figure 7 – how many points are in each topographic region ? Are points evenly distributed throughout ? | We thank the reviewer for this valuable suggestion regarding the spatial distribution of the geodetic control points. To address the concern, we have added a supplementary S1 figure showing the spatial distribution of the points across the study area. In addition, Tables 3-8 summarise the distribution of control points across land cover classes and topographic zones. We have acknowledged that one of the limitations of the study was inadequate points falling in the water category. We did not include this in our analysis (Lines 626-631). |
| For the water classed points, where do these fall within the DSM (DEM) pixels ? How much of it is shoreline vs waterbody ? You could inspect a sub-sample of the points to classify this component i.e. Geodetic control points (CPs) are on higher land near water, whereas the DSM pixel is mostly water. | The water land cover class has been excluded from the revised analysis due to the very small number of control points in this category, which limited the reliability of statistical inference as suggested by one reviewer.  We acknowledge that the water class raises additional interpretation challenges related to shoreline effects and mixed land–water pixels. |
| I don’t see an comparison of the horizontal offsets of the DSM (DEM) grids compared to the geodetic CPs. One method it to compare the pixel centre distance to the geodetic CPs – this will given an idea of how “central” the pixel grids are relative to the geodetic CPs and which are near edges of the pixel and/or notable elevation differences in pixel relative heights. For example how to the neighbouring pixels (3 x 3 window) relative the pixel elevation compared to the geodetic CPs. This may address some of the issues of outliers mentioned. | We thank the reviewer for this valuable suggestion. To assess the potential influence of local neighbourhood effects and sub-pixel positional uncertainty, we conducted a sensitivity analysis by comparing elevations extracted from the centre pixel with those derived from a 3×3 focal mean window. The resulting ME, MAE, RMSE and SD values were nearly identical for all three DSMs, indicating that neighbourhood averaging had a negligible effect on the reported accuracy statistics. These results suggest that local pixel-edge effects and minor horizontal offsets did not materially influence the observed elevation errors. The results have now been included in the revised manuscript (Supplementary S2 Table) (Lines 626-270, 335-338) |
| How do the results compare if you compare all the variables together ? In other words if you put all the variable together, topographic region AND land cover AND slope AND aspect, what is revealed ? For example are forested south aspect hills with slopes of greater than 10 % and elevations above 1300 m the least accurate ? Can you assess performance of the DSMs as a whole. | In the revised manuscript, we address this by applying a Random Forest (RF) model, which inherently captures non-linear relationships and interaction effects among predictor variables (Lines 301-322, 446-457) |
| What is the present land cover / land use of the of the Malawian “high-precision national geodetic control points” ? And at the time of the satellite surveys (e.g. 2000 and 2013) ? Have there been any significant changes in land use between the satellite surveys and present day ? This would help discern some of the difference between the Malawian geodetic CPs – how do those in urban settings compare to those in rural settings in terms of the various measures of performance against the satellite surveys ? Where any of these areas rural at the time of the satellite surveys but are now urban ? What is the impact on the results for those situations ? | To address temporal mismatch as far as possible, we now used the 30 m Global Land Cover Estimation (GLanCE) dataset and selected land cover products closest to the respective DEM acquisition periods (2001 for SRTM and NASADEM, and 2010 for ASTER GDEM). This approach provides a more representative approximation of land surface conditions at the time of satellite data acquisition. Although we acknowledge that it does not fully eliminate temporal inconsistencies. This is now explicitly stated as a limitation in the revised manuscript since the actual ground condition at the time of data capturing since images were captured several different years (Lines 209-219, 633-642). |
| Line 64-65 : “Interestingly, ASTER GDEM 65 and NASADEM performed better in dense forest areas [10].” This is contradicted by the statements in Line 66-67. Why do you consider this interesting ? Dense forests would be seen as a coherent surface and so ASTER GDEM and NASADEM, both being DSMs and having limited no (ASTER GDEM) to limited (NASADEM) penetration, and so would both “see” a similar elevation. Re-work paragraph. | We thank the reviewer for this valuable comment. We agree that the previous wording overstated the interpretation of the observed results. The paragraph has been fully revised to improve logical consistency and interpretation (Lines 66-67) |
| Line 72: Unsubstantiated statement - what other assessments have indicated that DSM (DEM) use is uncertain in Malawi or surround region ? | The sentence has been deleted |
| Line 77-78: refers to “cropland, grassland, forest reserves, built-up areas, and wetlands” which are different land cover classes given in Figure 1d. Harmonise the two sets of classes. | Thank you for the observation. This has been rectified |
| Line 188 – 191: What is the distribution of the 2 404 benchmark CPs ? Also see comment regarding DSM vs DEM/bare earth elevations. | The distribution of points across land cover classes and physiographic settings is presented in Tables 3-8 and Supplementary S1 Figure. The control points are broadly distributed across the study area, covering major land cover types and topographic zones, including lowland, mid-elevation, and highland regions. |
| Line 207-213 : did you compare the elevation histograms before and post resampling and reprojection ? How did the resampling affect the min, max and mean of elevations ? A table of this should be shown as a supplementary. | We thank the reviewer for this comment. To improve transparency of the preprocessing workflow, we have now included a summary table showing DEM statistics before and after resampling (Supplementary S1 Table). The results indicate that there are only negligible differences in minimum, maximum, mean, and standard deviation values across ASTER GDEM, SRTM, and NASADEM following resampling (lines 262-270). |
| Line 210: What resampling method was used ? This needs to be stated e.g. nearest neighbour, bilinear interpolation, cubic convolution etc as these will affect the distribution of the elevations, especially in different topographic settings. | We thank the reviewer for this important clarification. The manuscript has been updated to explicitly state that nearest neighbour resampling was used during the reprojection of all DEM datasets to a common coordinate reference system (Lines 227-235). |
| Line 212: How did you create the same reference grid for each DSM raster? And if it was not the same reference grid, what is the pixel offset between DSM raster grids? | We thank the reviewer for this comment. All DSMs were clipped, reprojected to a common coordinate system (WGS84 / UTM Zone 36L), and aligned to a common 30 m reference grid using nearest neighbour resampling and a snap-to-raster approach. This ensured consistent grid geometry (origin, resolution, and extent) across all datasets. We have now added this information (Lines 232-235) |
| Line 219-220: This point-by-point dataset needs to be shown as a supplementary table. | We thank the reviewer for this suggestion. We agree on the importance of data transparency. We have now made the data accessible from this link: [10.5281/zenodo.21101053](https://doi.org/10.5281/zenodo.21101053). |
| Line 222: For outlier removal, how did you determine that their were outliers to be removed ? And how many of these outliers were a result of resampling and/or pixel shifts and/or edge effects ? | We thank the reviewer for this comment. We clarify that no outlier removal or statistical filtering was applied in the revised manuscript. |
| Line 237-238: Why would there be a difference between the 3 DSM ? Why not just compare the same number of points ? ie. 2229 points so that the comparison of the 3 DSMs is the same ? What is the justification for using different points between the DSMs ? | In the revised work, we have not removed outliers. The original 2404 points have been applied to all DSMs. |
| Line 278-2292: What points were removed ? And where did they sit in the landscape ? Was it near the edges of Malawi ? Was it mostly near water or in mountain peaks ? Where important extreme features removed e.g. mountain tops and deep valley bottoms ? | No outlier filtering was applied in the revised manuscript. All geodetic control points were retained in the analysis for all DSMs |
| Line 329-330: What does the N between Aspect and ME in Table 3 mean ? Number of points analysed ? If so there is a +40 % difference in some of the point numbers assessed. How do you think this might have affected the analysis ? This is why knowing the geographic distribution of the points is important. | We thank the reviewer for this useful comment. We confirm that N in Table 3 represents the number of geodetic control points used in each aspect category for the analysis. The differences in sample size across aspect classes arise from the spatial distribution of the control points across the study area.  We acknowledge that variation in sample sizes may introduce some level of imbalance in the statistical comparison; however, given the large overall sample size and the broad spatial coverage of control points, we consider the results to remain robust. This limitation has now been explicitly stated in the revised manuscript (Lines 627-. |
| Line 362-363: 14 points is not a large amount – it s a below the “rule-of-thumb” of 30 data points needed for statistical analysis. Where were these points located ? On bridges ? Shorelines ? River gauges ? | We thank the reviewer for this observation. We agree that the number of points classified under water was insufficient for robust statistical inference. In the revised manuscript, these points have been excluded from the analysis to avoid unreliable estimates and potential bias in the statistical comparisons. This limitation has now been stated in the revised manuscript. |
| Line 373 : Topography is not only about elevation bands. Does slope curvature play a role here ? e.g. convex slops in the highlands have less error than convex slops in the highlands. Where on the land surface the point is matters as much as how high that surface is. | We thank the reviewer for this insightful comment. In response, curvature has now been incorporated into the revised analysis (Line 420, 627-631). |
| Line 376-377: What filtering is being referred to ? It seems like the 2333 points ? | In the revised manuscript, we have removed the filtering part. |
| Line 398-402: Did you check the NUM file (see [https://lpdaac.usgs.gov/documents/434/ ASTGTM_User_Guide_V3.pdf](https://lpdaac.usgs.gov/documents/434/ASTGTM_User_Guide_V3.pdf)) to assess how many scenes were used per a tile in Malawi ? Do some tiles have substantially less scenes then others ? As you indicate – this is an important component and therefore needs to be assessed. | We thank the reviewer for this insightful comment. We did not perform a full tile-by-tile quantitative analysis of the NUM file across Malawi. However, we acknowledge that spatial variations in scene count may exist and could influence local accuracy. The version 3 product already incorporates an increased number of scenes and improved stacking procedures compared to earlier versions. Nevertheless, we recognise that residual spatial variability in scene density may still contribute to differences in local accuracy.  This limitation has been acknowledged in the revised manuscript, and future work could explicitly incorporate NUM-layer analysis to further assess the relationship between scene density and elevation accuracy (Lines 644-651). |
| Line 429: Could the tree cover difference be a change in land cover between year of assessment and year of satellite survey ? In the case of SRTM is have been +20 years since the data was collected. Trees can go big. Further more in what direction are these elevations ? Positive or negative ? These changes could be assessed, and quantified to a degree using Global Forest Watch Data: https://www.globalforestwatch.org/map/ | We thank the reviewer for this insightful comment. **To minimise this effect**, we used land cover datasets closest to the respective DSM acquisition periods (2001 for SRTM and NASADEM, and 2010 for ASTER GDEM), rather than contemporary land cover. This approach reduces but does not fully eliminate temporal mismatch between vegetation conditions and DSM acquisition dates. We agree that products such as Global Forest Watch could provide further insight into vegetation change dynamics; however, this was beyond the scope of the present study and is suggested as a valuable direction for future work (Lines 633-642). |
| Line 432-433: You indicate that SRTM (and subsequently NASA DEM) is indeed a DSM and not DEM | We have revised the manuscript to consistently refer to ASTER GDEM, SRTM, and NASADEM as DSMs. We have also clarified the implications of this distinction in the interpretation of results, particularly that lower accuracy in vegetated areas may be may the nature of the products to include canopy rather than errors in the DSM products. |
| Figure 1: include coordinates on at least 1 panel. Label Lake Malawi in all panels. Scale bar should be displayed to nearest 50 km or 100 km, a 240 km scale bar is difficult to use.  Figure 1 (b): included Lake Malawi in the Legend – surely it is a topographic region in its own right ? Lake Chilwa should also be show and labelled. Up to authors if Lake Malombe should be displayed. | Figure 1 has been revised accordingly. Geographic coordinates have been added to panel. Lake Malawi is now labelled in all panels for consistency. The scale bar has been modified to 50km interval. Lake Malawi has been labelled on the map, as it is a geographic feature rather than a topographic region. Lake Chilwa and Malombe have also been added and labelled. |
| Figure 1 (c): Legend indicates a high of 408 % ? Slope ranges are 0 % (flat) to 90 % (vertical). Check legend labels or calculation. Lake Chilwa to be displayed | We thank the reviewer for this observation. We carefully checked the slope values in the dataset and confirmed that the steep slopes identified in the analysis are correct. These high slope values are predominantly located in the Mulanje Mountain area in southern Malawi. The highest peak in south-central Africa. |
| Figure 1 (d): Land use / Land cover legend shows clouds. This is not a Land Cover type. Correct legend. At scale of map difficult to see difference in Crop and Rangeland as colours similar. I suggest changing the colour e.g. crops to purple. | The map has been revised accordingly. |
| REVIEWER 2 |  |
| 1.1 Broken / Duplicated Sentence at Table 2 Callout  The sentence introducing Table 2 in the Results section is malformed and split across two paragraphs:  "The vertical accuracy assessment revealed notable differences among the three DEMs ( ASTER, NASADEM, and SRTM underestimated the true ground elevations by -7.03 m, -5.86, and -4.56, respectively. Table 2). Before filtering…"  The underestimation values (-7.03 m, -5.86 m, -4.56 m) are then repeated verbatim at the end of the same paragraph. This appears to be a copy-paste error and makes the Results section confusing. The sentence should be restructured so the Table 2 callout appears naturally and the duplicate text is removed. | We thank the reviewer for identifying this issue. This has now been corrected in the revised manuscript by restructuring the sentence for clarity and removing the repeated text. |
| Typo 1: "STRM" should be "SRTM" — "the present study evaluated how well STRM, ASTER, and NASADEM…" (line 94)  Typo 2: "NASASEM" should be "NASADEM" — "Our findings that NASASEM outperformed both SRTM and ASTER…" (line 411) | Spelling mistakes have been corrected in the revised manuscript. Thank you for the observation. |
| 1.3 Inconsistent Citation Style The manuscript uses a numbered citation style throughout (e.g., [1], [10], [42]). However, two inline author-year citations appear without a corresponding numbered entry in the reference list: "(Huang & Yu, 2024)" — line 79. No numbered reference exists for this. "(Sun et al., 2003)" — line 159. No numbered reference exists for this. Both must be converted to the numbered format and added to the reference list. | We thank the reviewer for identifying this inconsistency. The citations have been corrected accordingly. |
| 2.1 No Vertical Datum Reconciliation (Critical) SRTM, NASADEM, and ASTER GDEM V3 heights are all referenced to the EGM96 geoid. The national geodetic benchmarks in Malawi likely reference a local vertical datum tied to a local mean sea level or national height system. The paper does not mention any geoid correction or vertical datum transformation when comparing DEM-derived elevations to benchmark elevations. This is a fundamental methodological gap. Failing to reconcile vertical datums can introduce systematic biases that inflate or deflate all reported RMSE and ME values, undermining the validity of the accuracy assessment. The authors must explicitly state the vertical datum of the benchmarks and describe any transformation applied (or justify why none was needed). | We thank the reviewer for this critical observation. We agree that vertical datum consistency is essential for reliable DSM accuracy assessment.  In the revised manuscript, we have clarified and addressed this issue. The national geodetic control points in Malawi were originally referenced to a local vertical datum tied to mean sea level observations from the Beira tide gauge (Mozambique). To ensure consistency with the global DSM products, which are referenced to the EGM96 geoid, the benchmark elevations have now been transformed to the EGM96 vertical datum using a defined vertical offset model (Lines 197-207) |
| 2.2 SRTM Version Inconsistency  The paper states that "SRTM DEM version 4.1" was used — this refers to the CGIAR-CSI void-filled product. However, the paper also states it was downloaded from the USGS Earth Explorer portal, which distributes the NASA/USGS 1 Arc-Second Global SRTM, not the CGIAR v4.1 product. These are different products with different void-filling algorithms and processing chains. The authors must clarify exactly which SRTM product was used, from which source, and ensure the description is internally consistent. | Thank you for the observation. In the revised work, we have now used SRTM version 3.0 downloaded from the NASA Earthdata. |
| 2.3 Circular Dependency: Slope and Aspect Derived from the DEMs Under Evaluation  Terrain derivatives (slope and aspect) used to stratify the accuracy analysis were computed from each DEM using ArcGIS. This creates a circular dependency: the independent variable used to explain DEM error is itself derived from the DEM being assessed. Ideally, slope and aspect should be computed from an independent, higher-accuracy reference DEM (e.g., a LiDAR-derived DTM or a higher-resolution national product). At minimum, this limitation must be clearly acknowledged and its potential influence on results discussed. | We thank the reviewer for this important methodological observation. We acknowledge this as a limitation, and we have explicitly stated in the revised manuscript that terrain derivatives derived from the evaluated DSMs may partially reflect characteristics of the same data used in the error analysis (Lines 157-162) |
| 3.1 Critically Small Sample Sizes in Key Strata (Critical)  Several of the paper's key conclusions are drawn from strata with very small sample sizes:  Steep slopes (>30°): n = 45 (ASTER), 23 (SRTM), 22 (NASADEM). Drawing conclusions about DEM accuracy on steep terrain — one of the central findings of the paper — from only 22–45 points is statistically unreliable.  Water land cover: n = 14 across all three DEMs. Results for this category should be excluded or accompanied by a strong caveat that sample size precludes interpretation.  Trees land cover: n = 59–64. Also low for a category central to the land cover analysis.  The paper should quantify the spatial distribution of reference points across strata and report confidence intervals for accuracy metrics in small-sample categories. Conclusions drawn from n < 30 must be explicitly caveated. | We thank the reviewer for this important comment. We agree that small sample sizes can limit the reliability of statistical inference. In response, the water land cover category (n = 14) has been removed from the revised analysis.  For the remaining categories, the number of reference points reflects the actual spatial distribution of the national geodetic control network across Malawi. In particular, steep slopes (>30°) and tree cover occupy relatively limited areas where geodetic benchmarks are available, resulting in smaller sample sizes. Rather than excluding these categories, we have retained them because they represent important landscape conditions for DSM evaluation. However, we have revised the manuscript to explicitly acknowledge that conclusions for these classes should be interpreted with caution due to the limited number of observations (Lines 589-591, 627-631). |
| 3.2 No Statistical Significance Testing for Inter-DEM Comparisons (Critical)  The paper concludes that NASADEM outperforms SRTM, yet the difference between their national-scale RMSEs is only 0.13 m (16.69 vs. 16.82 m). No paired statistical tests (e.g., Wilcoxon signed-rank test, paired t-test on residuals, or bootstrapped confidence intervals) are performed to determine whether differences between DEMs are statistically significant. Without significance testing, ranking the three DEMs by performance is not rigorously justified, particularly for NASADEM vs. SRTM where differences are marginal. | We thank the reviewer for this important observation. In response, we have incorporated both independent-sample t-tests and Wilcoxon rank-sum tests. We performed for each DSM pair, and Cohen's d was calculated to quantify the magnitude of the observed differences (supplementary S3 Table)(Lines 288-299). |
| 3.3 Different Point Sets After IQR Filtering Reduce Comparability The IQR filter was applied independently to each DEM, yielding different subsets of reference points (n = 2,233 for ASTER; 2,232 for NASADEM; 2,229 for SRTM). Comparisons across DEMs are therefore not made on the same set of reference points, which introduces a confounding factor. Ideally, either the union or intersection of the three filtered point sets should be used for cross-DEM comparisons, or the approach should be clearly justified. | We thank the reviewer for this important observation. We agree that applying independent filtering to each DSM could reduce the comparability of the results.  In the revised manuscript, no IQR-based outlier filtering was performed. Instead, the complete set of 2,404 geodetic control points was used consistently for all DSMs in the revised work. |
| 3.4 Misleading Use of R² as Evidence of Accuracy Lines 299–302 state that R² = 0.997–0.998 in the DEM-vs-reference regression indicates the DEMs "closely track reference elevations with minimal bias." This interpretation is misleading. A high R² is expected whenever both variables span a large dynamic range (Malawi ranges from near sea level to ~3,000 m). R² reflects the proportion of variance explained and is heavily inflated by dynamic range; it does not indicate point-level accuracy. The RMSE (~17–18 m) is the appropriate accuracy measure. The R² claim should be removed or reframed to avoid overstating DEM performance. | We thank the reviewer for this important observation and agree that a high coefficient of determination (R²) should not be interpreted as evidence of high vertical accuracy.  In the revised manuscript, we have reworded the relevant section to clarify that the regression analysis is intended to assess the relationship between DSM elevations and the reference elevations. |
| 4. Content & Scientific Issues 4.1 Study Area Description Inconsistency Lines 109–112 state that Malawi is subdivided into "four main physiographic regions" but then proceed to list five: the Lower Shire Valley, the Lakeshore Plain, Middle and Upper Shire, Mid-elevation Plain, and the Highlands. This internal contradiction should be corrected. | We thank the reviewer for this observation. We clarify that there is no inconsistency in the physiographic classification used in the study.  The lake shore plain and middle and Upper Shire (200 m to 760 m) were intended to be treated as a single physiographic region, consistent with Malawi’s elevation-based landscape classification. However, the original wording may have created ambiguity by listing them separately. In the revised manuscript, the description has been rephrased for clarity (Lines 111-112). |
| 4.2 Misplaced Reference [18] Reference [18] (Wickham et al., 2023) is an accuracy assessment of the NLCD 2019 land cover product for the conterminous United States. It does not relate to DEM accuracy assessment and appears to have been incorrectly inserted into the DEM literature review. This reference should be removed or replaced with the intended citation. | Thank you for the observation. It has been removed. |
| 4.3 Incomplete Author Name in Reference [4] Reference [4] reads: "Sun, Li J. A method for extracting small water bodies based on DEM and remote sensing images. Sci Rep. 2024;14: 760." The first author's given name or initials are missing, leaving only "Sun," as the first author entry. This should be corrected. | It has been corrected (Line 667) |
| Reviewer #2: 1 – Partly : Refinement is required in light of the Digital Surface Model vs Digital Elevation Model, these concepts have important implications for the interpretation of the results. | We thank the reviewer for the observation. We agree that a clear distinction between Digital Surface Models (DSMs) and Digital Elevation Models (DEMs) is essential for correct interpretation of the results. In the revised manuscript, we have undertaken a comprehensive refinement to ensure conceptual consistency. |
| 2 – No : Consideration for differences of landcover affects at time of satellite survey vs Malawian geodectic control points not expounded on. Distribution of points is not given, so not possible to determine if there is an even distribution and comparable number of points across topographic regions, land cover types and aspect – how many points occur in each of the categories of these parameters ? | We thank the reviewer for this important comment. We agree that the spatial distribution of reference points across land cover, topographic regions, and aspect classes is important for interpreting DSM accuracy results.  In this study, the geodetic control points are not a statistically sampled dataset but represent the complete available national geodetic control network, which is primarily designed for surveying and cadastral purposes rather than stratified sampling. As a result, their distribution reflects the underlying placement of national benchmarks rather than an even sampling design. We also acknowledge in the revised Discussion that uneven spatial distribution of control points across certain terrain and land cover classes may influence the robustness of class-specific accuracy estimates, particularly in sparsely represented categories.  However, to improve transparency and reproducibility, we have now provided a Supplementary S1 Figure. In addition, the spatial distribution of the 2,404 control points across physiographic regions, land cover classes, and aspect categories is provided in Tables 3-8. |
| 3 – No: Elevations and locations of Malawian geodetic controls not provided, therefore the analysis cannot be replicated based on the data provided in paper. | We thank the reviewer for this important comment regarding reproducibility. We have now made the data accessible from this link: [10.5281/zenodo.21101053](https://doi.org/10.5281/zenodo.21101053). |
