# Supplementary material for: Vertical accuracy of ASTER, SRTM, and NASADEM digital surface models across Malawi’s diverse topography
Source: PLoS One. 2026 Oct 29;21(10):e0361397. doi: 10.1371/journal.pone.0361397 (PMC13718972; doi:10.1371/journal.pone.0361397)
Supplement: Supplementary file 3 [file pone.0361397.s005.docx]

Vertical accuracy of ASTER, SRTM, and NASA Digital Elevation Models across Malawi’s diverse topography

**Overall comment**

This manuscript has merit, and may provide an important reference for future work on the performance of DSMs/DEMs as well as choosing which DSM/DEM to use for other studies.

However it is currently constrained by some fundamental issues, namely:

a) ensuring that the distinction of DSM vs DEM and the interpretation of the results in such a distinction (.i.e. control points are bare earth elevations, SRTM is not a bare earth elevation). The issue is mentioned by the authors themselves in line 431 – 433, where it is pointed out that SRTM is a DSM (tree canopy is “seen”) but this issues is not dealt with during the analysis.

b) the presentation of all the data and techniques. In its present state I could not repeat the study as I do not have any information on the Malawian geodectic control points (or know who to contact to get them should they be considered sensitive); nor do I know how the DSMs were prepared prior to analysis (i.e. how they were resampled, how the were reprojected).

I am of the opinion that the two above points will substantially change the interpretation and discussion of the article. Especially in combination with the points and comments listed below.

**General comments**

*Comment 1*

Authors need to differentiate between digital surface model and digital terrain model as it has implications for the interpretation of their results. SRTM and NASADEM are both derived from RADAR interferometry, and do not represent a bare earth surface model (i.e. a “true” DEM); similarly ASTER GDEM is based on stereo scence. Consequently they are DSM, i.e. dense canopy and buildings contribute to the height values of individual cells. See for example, Jacobsen and Passini (2010) and Simard et al. (2024)

*Comment 2*

Supplementary table of points assess I need.

e.g. Point Name, Lat, Long, Control Point Elevation, DEM 1 Elevation, DEM 2 Elevation, DEM 3 Elevation

These points should also be shown a on Supplementary map.

Reason is that not indication of spread of points is given, there may be a bias in spatial distribution of points that may influence the result. Such point geographic distribution needs to be addressed in text. For example, where are the “Water” land cover type (Figure 6) points located ?

Similar for Figure 7 – how many points are in each topographic region ? Are points evenly distributed throughout ?

*Comment 3*

For the water classed points, where do these fall within the DSM (DEM) pixels ? How much of it is shoreline vs waterbody ? You could inspect a sub-sample of the points to classify this component i.e. Geodetic control points (CPs) are on higher land near water, whereas the DSM pixel is mostly water.

*Comment 4*

I don’t see an comparison of the horizontal offsets of the DSM (DEM) grids compared to the geodetic CPs. One method it to compare the pixel centre distance to the geodetic CPs – this will given an idea of how “central” the pixel grids are relative to the geodetic CPs and which are near edges of the pixel and/or notable elevation differences in pixel relative heights. For example how to the neighbouring pixels (3 x 3 window) relative the pixel elevation compared to the geodetic CPs. This may address some of the issues of outliers mentioned.

*Comment 5*

How do the results compare if you compare all the variables together ? In other words if you put all the variable together, topographic region AND land cover AND slope AND aspect, what is revealed ? For example are forested south aspect hills with slopes of greater than 10 % and elevations above 1300 m the least accurate ? Can you assess performance of the DSMs as a whole.

**Specific Content comments**

What is the present land cover / land use of the of the Malawian “high-precision national geodetic control points” ? And at the time of the satellite surveys (e.g. 2000 and 2013) ? Have there been any significant changes in land use between the satellite surveys and present day ? This would help discern some of the difference between the Malawian geodetic CPs – how do those in urban settings compare to those in rural settings in terms of the various measures of performance against the satellite surveys ? Where any of these areas rural at the time of the satellite surveys but are now urban ? What is the impact on the results for those situations ?

Line 64-65 *: “Interestingly, ASTER GDEM 65 and NASADEM performed better in dense forest areas [10].”* This is contradicted by the statements in Line 66-67. Why do you consider this interesting ? Dense forests would be seen as a coherent surface and so ASTER GDEM and NASADEM, both being DSMs and having limited no (ASTER GDEM) to limited (NASADEM) penetration, and so would both “see” a similar elevation. Re-work paragraph.

Line 72: Unsubstantiated statement - what other assessments have indicated that DSM (DEM) use is uncertain in Malawi or surround region ?

Line 77-78: refers to *“cropland, grassland, forest reserves, built-up areas, and wetlands”* which are different land cover classes given in Figure 1d. Harmonise the two sets of classes.

Line 188 – 191: What is the distribution of the 2 404 benchmark CPs ? Also see comment regarding DSM vs DEM/bare earth elevations.

Line 207-213 : did you compare the elevation histograms before and post resampling and reprojection ? How did the resampling affect the min, max and mean of elevations ? A table of this should be shown as a supplementary.

Line 210: What resampling method was used ? This needs to be stated e.g. nearest neighbour, bilinear interpolation, cubic convolution etc as these will affect the distribution of the elevations, especially in different topographic settings.

Line 212: How did you create the same reference grid for each DSM raster ? And if it was not eh same reference grid, what is the pixel offset between DSM raster grids ?

Line 219-220: This point-by-point dataset needs to be shown as a supplementary table.

Line 222: For outlier removal, how did you determine that their were outliers to be removed ? And how many of these outliers were a result of resampling and/or pixel shifts and/or edge effects ?

Line 237-238: Why would there be a difference between the 3 DSM ? Why not just compare the same number of points ? ie. 2229 points so that the comparison of the 3 DSMs is the same ? What is the justification for using different points between the DSMs ?

Line 278-2292: What points were removed ? And where did they sit in the landscape ? Was it near the edges of Malawi ? Was it mostly near water or in mountain peaks ? Where important extreme features removed e.g. mountain tops and deep valley bottoms ?

Line 329-330: What does the N between Aspect and ME in Table 3 mean ? Number of points analysed ? If so there is a +40 % difference in some of the point numbers assessed. How do you think this might have affected the analysis ? This is why knowing the geographic distribution of the points is important.

Line 362-363: 14 points is not a large amount – it s a below the “rule-of-thumb” of 30 data points needed for statistical analysis. Where were these points located ? On bridges ? Shorelines ? River gauges ?

Line 373 : Topography is not only about elevation bands. Does slope curvature play a role here ? e.g. convex slops in the highlands have less error than convex slops in the highlands. Where on the land surface the point is matters as much as how high that surface is.

Line 376-377: What filtering is being referred to ? It seems like the 2333 points ?

Line 398-402: Did you check the NUM file (see [https://lpdaac.usgs.gov/documents/434/
ASTGTM_User_Guide_V3.pdf](https://lpdaac.usgs.gov/documents/434/ASTGTM_User_Guide_V3.pdf)) to assess how many scenes were used per a tile in Malawi ? Do some tiles have substantially less scenes then others ? As you indicate – this is an important component and therefore needs to be assessed.

Line 429: Could the tree cover difference be a change in land cover between year of assessment and year of satellite survey ? In the case of SRTM is have been +20 years since the data was collected. Trees can go big. Further more in what direction are these elevations ? Positive or negative ? These changes could be assessed, and quantified to a degree using Global Forest Watch Data: https://www.globalforestwatch.org/map/

Line 432-433: You indicate that SRTM (and subsequently NASA DEM) is indeed a DSM and not DEM

Figure 1: include coordinates on at least 1 panel. Label Lake Malawi in all panels. Scale bar should be displayed to nearest 50 km or 100 km, a 240 km scale bar is difficult to use.

Figure 1 (b): included Lake Malawi in the Legend – surely it is a topographic region in its own right ? Lake Chilwa should also be show and labelled. Up to authors if Lake Malombe should be displayed.

Figure 1 (c): Legend indicates a high of 408 % ? Slope ranges are 0 % (flat) to 90 % (vertical). Check legend labels or calculation. Lake Chilwa to be displayed

Figure 1 (d): Land use / Land cover legend shows clouds. This is not a Land Cover type. Correct legend. At scale of map difficult to see difference in Crop and Rangeland as colours similar. I suggest changing the colour e.g. crops to purple.

**Specific Language and Writing comments**

The writing and the language of the paper is clear and correct.

**References**

Simard, M., Denbina, M., Marshak, C., & Neumann, M. (2024). A global evaluation of radar‐derived digital elevation models: SRTM, NASADEM, and GLO‐30. Journal of Geophysical Research: Biogeosciences, 129, e2023JG007672.https://doi.org/10.1029/2023JG007672

Jacobsen, K. and Passini, R., (2010). Analsysis of aster gdem elevation models. In *International Archives of the Photogrammetry, Remote Sensing and Spatial Information Sciences:[2010 Canadian Geomatics Conference And Symposium Of Commission I, ISPRS Convergence In Geomatics-Shaping Canada's Competitive Landscape] 38 (2010), Nr. Part 1* (Vol. 38, No. Part 1). Göttingen: Copernicus GmbH. <https://www.isprs.org/proceedings/xxxviii/part1/09/09_03_Paper_103.pdf>
