# Supplementary material for: Vertical accuracy of ASTER, SRTM, and NASADEM digital surface models across Malawi’s diverse topography
Source: PLoS One. 2026 Oct 29;21(10):e0361397. doi: 10.1371/journal.pone.0361397 (PMC13718972; doi:10.1371/journal.pone.0361397)
Supplement: Supplementary file 4 [file pone.0361397.s008.docx]

**Response to Reviewers**

| **Reviewer 1** |  |
| --- | --- |
| **Comments** | **Response** |
| Vertical datum transformation is described but not reproducible. Please report the numerical value of Δv in the EGM96 alignment equation, whether it is a constant or a spatially varying offset, and the source used to obtain it. | Thank you for this comment. We have clarified the vertical datum transformation procedure in the revised manuscript. The original benchmark elevations were referenced to local Mean Sea Level (MSL), tied to the Beira tide gauge in Mozambique. A mean offset of +1.01 m between the local MSL datum and EGM2008 was applied to convert the benchmark elevations to EGM2008. The subsequent conversion from EGM2008 to EGM96 was performed using point-specific differences between the two geoid models obtained from a geoid calculator. The revised manuscript now clearly distinguishes these two transformation steps and explains how the final EGM96 reference elevations were obtained (Lines 220-234). |
| Please state when the national geodetic control points were surveyed or last updated (even an approximate establishment period is sufficient). | Thank you for this comment. We have added information on the establishment period of the national geodetic control network. The Malawi geodetic control network was developed progressively between 1948 and 1974. The primary triangulation framework was established mainly during 1948-1953 and subsequent extensions and densification completed through 1974 (Lines 207-209). |
| Methods and the response letter describe independent-sample t-tests and Wilcoxon rank-sum tests, whereas Supplementary Table S3 is captioned as paired tests. Because the same 2,404 locations are used for all three DSMs, paired tests are the correct choice. Please make the Methods, Results, and Table S3 consistent. Relatedly, the national RMSE difference between SRTM (19.55 m) and NASADEM (19.99 m) is 0.44 m and should not be described as “substantially” lower. | Thank you for pointing this out. We have revised the statistical analysis description throughout the manuscript to consistently use paired tests because the same 2,404 geodetic control locations were used to evaluate all three DSMs. The Methods and Results now refer to paired t-tests and Wilcoxon signed-rank tests (Lines 315-326). We also revised statements implying large practical differences between SRTM and NASADEM and now describe the observed national RMSE difference (0.44 m) as small despite statistical significance (Lines 495-498 and 643-644). |
| The Abstract reports NASADEM RMSEs of 2.36–35.03 m. In Table 7, 2.36 m is the MAE for the Lower Shire Valley; the corresponding RMSE is 3.25 m. Please correct this. | We thank the reviewer for identifying this error. The Abstract has been corrected so that NASADEM RMSE values are now reported as 3.25 m (Line 33) |
| High R² (0.9966–0.9971) should not be presented as evidence of vertical accuracy; it is expected given Malawi’s large elevation range. RMSE and MAE remain the appropriate accuracy metrics. Please add this caveat in the Results. | We thank the reviewer for this important clarification. We agree that very high R² values largely reflect the broad elevation range of Malawi rather than DSM vertical accuracy. A caveat has now been added to the Results section explaining that RMSE, MAE, and ME remain the primary measures of vertical accuracy (Lines 373-378). |
| “(Huang & Yu, 2024)” remains as an author–year citation in the Introduction and is still missing from the numbered reference list. Please convert it to the journal’s numbered style. | We thank the reviewer for noting this oversight. The author-year citation has now been converted to the journal's numbered citation format and the full reference has been added to the reference list (lines 86 and 837). |
| **Reviewer 3** |  |
| The main finding of this study should be stated in abstract | Thank you for this suggestion. The Abstract has been revised to explicitly state the principal finding of the study. In particular, we now emphasise that DSM accuracy is strongly terrain-dependent and that national-scale accuracy statistics can conceal substantial spatial variation. We also report the contrasting performance between the Lower Shire Valley and the Highlands. |
| The resolution of all figures should be added | Thank you. All figures have been reviewed and prepared at publication-quality resolution using NAAS as recommended by the publisher. |
| All Tables should be Scientific Tables | All tables have been reformatted using a consistent scientific table format. |
| The necessity and innovation of the article should be presented to the introduction. | The Introduction has been substantially revised to clarify the research gap, necessity and innovation of the study. We now explain that national-scale accuracy statistics may conceal substantial spatial differences in DSM performance and explicitly state that the central argument of the study is that DSM suitability should be evaluated as a terrain-dependent property (lines 97-106). |
| It is suggested to compare the results of the present research with some similar studies which is done before. Refer add this findings  https://doi.org/10.1080/10298436.2025.2496335  https://doi.org/10.1016/j.triboint.2025.111348  https://doi.org/10.1007/s13131-025-2540-y  https://doi.org/10.1016/j.jhydrol.2025.133225  https://doi.org/10.1016/j.jhydrol.2025.132897  https://doi.org/10.1016/j.envsoft.2024.106138  https://doi.org/10.1016/j.rse.2025.115140  https://doi.org/10.1029/2025JC023214  https://doi.org/10.1016/j.jhydrol.2019.124516  https://doi.org/10.1002/nag.70323 | We carefully evaluated the suggested references and incorporated relevant papers where it directly supports the objectives of this study. |
| Much more explanations and interpretations must be added for the results, which are not enough and the discussions should be improved by comparing with previous studies.  https://doi.org/10.1016/j.enggeo.2026.108726  https://doi.org/10.1029/2026JE009657  10.1109/TGRS.2026.3654987  10.1130/B38406.1  https://doi.org/10.1016/j.ijmst.2026.01.008  https://doi.org/10.1016/j.ast.2026.113256  https://doi.org/10.3390/rs18142420  https://doi.org/10.12989/gae.2026.46.2.141  10.1007/s00603-025-05154-8  https://doi.org/10.1016/j.jvcir.2018.11.041 | We have substantially expanded both the Results and Discussion sections. The revised manuscript provides greater interpretation of the national-scale accuracy statistics, systematic elevation bias, terrain-dependent errors and land-cover effects (Lines 551-515, 517-519, 532-536, 593-597) |
| The major weakness of this study is that the debate/argument is not clearly articulated in the introduction. As a result, the paper’s contribution appears limited. I recommend that the author strengthen the theoretical discussion and explicitly develop the central debate/argument. | We agree with this observation. The Introduction has been revised to explicitly articulate the central scientific argument of the study: that global DSM accuracy should not be treated as spatially uniform and that national-scale accuracy statistics may conceal substantial terrain-dependent uncertainty. The Discussion has also been reorganised to develop this argument using the observed differences among Malawi's physiographic regions. In particular, the small national RMSE difference between SRTM and NASADEM is contrasted with the much larger differences observed between lowland and highland environments (lines 641-653). |
| Please ensure that the conclusions section clearly highlights the scientific value added of the paper and/or the practical applicability of your findings, as previously indicated. Revise the conclusion to include more detail—specifically, emphasise your key contributions, discuss the study’s limitations, underscore the scientific value of the results, and outline implications for future research. | The Conclusion has been substantially revised. It now clearly states the scientific contribution of demonstrating terrain-dependent DSM accuracy, summarises the major national and physiographic findings, discusses practical implications for hydrological, environmental, land-use and infrastructure applications, and identifies the main study limitations. |
|  |  |
