# Supplementary material for: Vertical accuracy of ASTER, SRTM, and NASADEM digital surface models across Malawi’s diverse topography
Source: PLoS One. 2026 Oct 29;21(10):e0361397. doi: 10.1371/journal.pone.0361397 (PMC13718972; doi:10.1371/journal.pone.0361397)
Supplement: Peer review history

# Peer review: Decision Letter 0

**DOI:** 10.1371/journal.pone.0361397.r001

**Authors:** Guy Schumann

6 May 2026

Dear Dr. Nazombe,

Please submit your revised manuscript by Jun 20 2026 11:59PM. If you will need more time than this to complete your revisions, please reply to this message or contact the journal office at plosone@plos.org. When you're ready to submit your revision, log on to https://www.editorialmanager.com/pone/ and select the 'Submissions Needing Revision' folder to locate your manuscript file.

- A letter that responds to each point raised by the academic editor and reviewer(s). You should upload this letter as a separate file labeled 'Response to Reviewers'.
- A marked-up copy of your manuscript that highlights changes made to the original version. You should upload this as a separate file labeled 'Revised Manuscript with Track Changes'.
- An unmarked version of your revised paper without tracked changes. You should upload this as a separate file labeled 'Manuscript'.

If you would like to make changes to your financial disclosure, please include your updated statement in your cover letter. Guidelines for resubmitting your figure files are available below the reviewer comments at the end of this letter.

If applicable, we recommend that you deposit your laboratory protocols in protocols.io to enhance the reproducibility of your results. Protocols.io assigns your protocol its own identifier (DOI) so that it can be cited independently in the future. For instructions see: https://journals.plos.org/plosone/s/submission-guidelines#loc-laboratory-protocols. Additionally, PLOS ONE offers an option for publishing peer-reviewed Lab Protocol articles, which describe protocols hosted on protocols.io. Read more information on sharing protocols at https://plos.org/protocols?utm_medium=editorial-email&utm_source=authorletters&utm_campaign=protocols.

As the corresponding author, your ORCID iD is verified in the submission system and will appear in the published article. PLOS supports the use of ORCID, and we encourage all coauthors to register for an ORCID iD and use it as well. Please encourage your coauthors to verify their ORCID iD within the submission system before final acceptance, as unverified ORCID iDs will not appear in the published article. *Only*  the individual author can complete the verification step; PLOS staff *cannot*  verify ORCID iDs on behalf of authors.

We look forward to receiving your revised manuscript.

Kind regards,

Guy J-P. Schumann

Section Editor

PLOS One

Journal Requirements:

When submitting your revision, we need you to address these additional requirements.

1. Please ensure that your manuscript meets PLOS ONE's style requirements, including those for file naming. The PLOS ONE style templates can be found at

https://journals.plos.org/plosone/s/file?id=wjVg/PLOSOne_formatting_sample_main_body.pdf and

https://journals.plos.org/plosone/s/file?id=ba62/PLOSOne_formatting_sample_title_authors_affiliations.pdf

2. Please note that PLOS One has specific guidelines on code sharing for submissions in which author-generated code underpins the findings in the manuscript. In these cases, we expect all author-generated code to be made available without restrictions upon publication of the work. Please review our guidelines at https://journals.plos.org/plosone/s/materials-and-software-sharing#loc-sharing-code and ensure that your code is shared in a way that follows best practice and facilitates reproducibility and reuse.

3. Please note that your Data Availability Statement is currently missing the DOI/accession number of each dataset OR a direct link to access each database. If your manuscript is accepted for publication, you will be asked to provide these details on a very short timeline. We therefore suggest that you provide this information now, though we will not hold up the peer review process if you are unable.

4. If the reviewer comments include a recommendation to cite specific previously published works, please review and evaluate these publications to determine whether they are relevant and should be cited. There is no requirement to cite these works unless the editor has indicated otherwise.

Additional Editor Comments:

In my opinion, the authors have an interesting paper here but it needs major revisions based on the comments received by reviewers. There are a number of excellent constructive comments, so I'd recommend the authors take care of all the comments received point-by-point.

[Note: HTML markup is below. Please do not edit.]

Reviewers' comments:

Reviewer's Responses to Questions

**Comments to the Author**

1. Is the manuscript technically sound, and do the data support the conclusions?

Reviewer #1: Yes

Reviewer #2: Partly

**********

2. Has the statistical analysis been performed appropriately and rigorously?

Reviewer #1: No

Reviewer #2: No

**********

3. Have the authors made all data underlying the findings in their manuscript fully available?

The PLOS Data policy

Reviewer #1: Yes

Reviewer #2: No

**********

4. Is the manuscript presented in an intelligible fashion and written in standard English?

Reviewer #1: Yes

Reviewer #2: Yes

**********

Reviewer #1: Overall Assessment

The paper addresses a genuine gap in the literature — no prior DEM vertical accuracy validation has been conducted in Malawi — and the study design is broadly sound. The use of 2,404 national geodetic benchmarks distributed across diverse topographic regions provides a credible reference dataset, and the comparison of three widely used global DEMs (ASTER GDEM V3, SRTM v4.1, NASADEM) across elevation, slope, aspect, and land cover strata is well-structured. However, there are several critical issues spanning methodology, statistics, writing, and reporting that must be addressed before the paper can be accepted for publication.

1. Writing & Formatting Errors

1.1 Broken / Duplicated Sentence at Table 2 Callout

The sentence introducing Table 2 in the Results section is malformed and split across two paragraphs:

"The vertical accuracy assessment revealed notable differences among the three DEMs ( ASTER, NASADEM, and SRTM underestimated the true ground elevations by -7.03 m, -5.86, and -4.56, respectively. Table 2). Before filtering…"

The underestimation values (-7.03 m, -5.86 m, -4.56 m) are then repeated verbatim at the end of the same paragraph. This appears to be a copy-paste error and makes the Results section confusing. The sentence should be restructured so the Table 2 callout appears naturally and the duplicate text is removed.

1.2 Typos

Typo 1: "STRM" should be "SRTM" — "the present study evaluated how well STRM, ASTER, and NASADEM…" (line 94)

Typo 2: "NASASEM" should be "NASADEM" — "Our findings that NASASEM outperformed both SRTM and ASTER…" (line 411)

1.3 Inconsistent Citation Style

The manuscript uses a numbered citation style throughout (e.g., [1], [10], [42]). However, two inline author-year citations appear without a corresponding numbered entry in the reference list:

"(Huang & Yu, 2024)" — line 79. No numbered reference exists for this.

"(Sun et al., 2003)" — line 159. No numbered reference exists for this.

Both must be converted to the numbered format and added to the reference list.

2. Methodological Issues

2.1 No Vertical Datum Reconciliation (Critical)

SRTM, NASADEM, and ASTER GDEM V3 heights are all referenced to the EGM96 geoid. The national geodetic benchmarks in Malawi likely reference a local vertical datum tied to a local mean sea level or national height system. The paper does not mention any geoid correction or vertical datum transformation when comparing DEM-derived elevations to benchmark elevations. This is a fundamental methodological gap. Failing to reconcile vertical datums can introduce systematic biases that inflate or deflate all reported RMSE and ME values, undermining the validity of the accuracy assessment. The authors must explicitly state the vertical datum of the benchmarks and describe any transformation applied (or justify why none was needed).

2.2 SRTM Version Inconsistency

The paper states that "SRTM DEM version 4.1" was used — this refers to the CGIAR-CSI void-filled product. However, the paper also states it was downloaded from the USGS Earth Explorer portal, which distributes the NASA/USGS 1 Arc-Second Global SRTM, not the CGIAR v4.1 product. These are different products with different void-filling algorithms and processing chains. The authors must clarify exactly which SRTM product was used, from which source, and ensure the description is internally consistent.

2.3 Circular Dependency: Slope and Aspect Derived from the DEMs Under Evaluation

Terrain derivatives (slope and aspect) used to stratify the accuracy analysis were computed from each DEM using ArcGIS. This creates a circular dependency: the independent variable used to explain DEM error is itself derived from the DEM being assessed. Ideally, slope and aspect should be computed from an independent, higher-accuracy reference DEM (e.g., a LiDAR-derived DTM or a higher-resolution national product). At minimum, this limitation must be clearly acknowledged and its potential influence on results discussed.

3. Statistical & Analytical Issues

3.1 Critically Small Sample Sizes in Key Strata (Critical)

Several of the paper's key conclusions are drawn from strata with very small sample sizes:

Steep slopes (>30°): n = 45 (ASTER), 23 (SRTM), 22 (NASADEM). Drawing conclusions about DEM accuracy on steep terrain — one of the central findings of the paper — from only 22–45 points is statistically unreliable.

Water land cover: n = 14 across all three DEMs. Results for this category should be excluded or accompanied by a strong caveat that sample size precludes interpretation.

Trees land cover: n = 59–64. Also low for a category central to the land cover analysis.

The paper should quantify the spatial distribution of reference points across strata and report confidence intervals for accuracy metrics in small-sample categories. Conclusions drawn from n < 30 must be explicitly caveated.

3.2 No Statistical Significance Testing for Inter-DEM Comparisons (Critical)

The paper concludes that NASADEM outperforms SRTM, yet the difference between their national-scale RMSEs is only 0.13 m (16.69 vs. 16.82 m). No paired statistical tests (e.g., Wilcoxon signed-rank test, paired t-test on residuals, or bootstrapped confidence intervals) are performed to determine whether differences between DEMs are statistically significant. Without significance testing, ranking the three DEMs by performance is not rigorously justified, particularly for NASADEM vs. SRTM where differences are marginal.

3.3 Different Point Sets After IQR Filtering Reduce Comparability

The IQR filter was applied independently to each DEM, yielding different subsets of reference points (n = 2,233 for ASTER; 2,232 for NASADEM; 2,229 for SRTM). Comparisons across DEMs are therefore not made on the same set of reference points, which introduces a confounding factor. Ideally, either the union or intersection of the three filtered point sets should be used for cross-DEM comparisons, or the approach should be clearly justified.

3.4 Misleading Use of R² as Evidence of Accuracy

Lines 299–302 state that R² = 0.997–0.998 in the DEM-vs-reference regression indicates the DEMs "closely track reference elevations with minimal bias." This interpretation is misleading. A high R² is expected whenever both variables span a large dynamic range (Malawi ranges from near sea level to ~3,000 m). R² reflects the proportion of variance explained and is heavily inflated by dynamic range; it does not indicate point-level accuracy. The RMSE (~17–18 m) is the appropriate accuracy measure. The R² claim should be removed or reframed to avoid overstating DEM performance.

4. Content & Scientific Issues

4.1 Study Area Description Inconsistency

Lines 109–112 state that Malawi is subdivided into "four main physiographic regions" but then proceed to list five: the Lower Shire Valley, the Lakeshore Plain, Middle and Upper Shire, Mid-elevation Plain, and the Highlands. This internal contradiction should be corrected.

4.2 Misplaced Reference [18]

Reference [18] (Wickham et al., 2023) is an accuracy assessment of the NLCD 2019 land cover product for the conterminous United States. It does not relate to DEM accuracy assessment and appears to have been incorrectly inserted into the DEM literature review. This reference should be removed or replaced with the intended citation.

4.3 Incomplete Author Name in Reference [4]

Reference [4] reads: "Sun, Li J. A method for extracting small water bodies based on DEM and remote sensing images. Sci Rep. 2024;14: 760." The first author's given name or initials are missing, leaving only "Sun," as the first author entry. This should be corrected.

Reviewer #2: 1 – Partly : Refinement is required in light of the Digital Surface Model vs Digital Elevation Model, these concepts have important implications for the interpretation of the results.

2 – No : Consideration for differences of landcover affects at time of satellite survey vs Malawian geodectic control points not expounded on. Distribution of points is not given, so not possible to determine if there is an even distribution and comparable number of points across topographic regions, land cover types and aspect – how many points occur in each of the categories of these parameters ?

3 – No: Elevations and locations of Malawian geodetic controls not provided, therefore the analysis cannot be replicated based on the data provided in paper.

4 – Yes : The writing and the language of the paper is clear and correct.

**********

what does this mean?). If published, this will include your full peer review and any attached files.

If you choose “no”, your identity will remain anonymous but your review may still be made public.

**Do you want your identity to be public for this peer review?**  For information about this choice, including consent withdrawal, please see our Privacy Policy

Reviewer #1: No

Reviewer #2: No

**********

[NOTE: If reviewer comments were submitted as an attachment file, they will be attached to this email and accessible via the submission site. Please log into your account, locate the manuscript record, and check for the action link "View Attachments". If this link does not appear, there are no attachment files.]

To ensure your figures meet our technical requirements, please review our figure guidelines: https://journals.plos.org/plosone/s/figures

You may also use PLOS’s free figure tool, NAAS, to help you prepare publication quality figures: https://journals.plos.org/plosone/s/figures#loc-tools-for-figure-preparation.

NAAS will assess whether your figures meet our technical requirements by comparing each figure against our figure specifications.

Attachment Submitted filename: PLOS_One_review.docx

# Author comment: Author response to Decision Letter 1

**DOI:** 10.1371/journal.pone.0361397.r002

3 Jul 2026

We have carefully revised the manuscript to ensure that it complies with the PLOS ONE style requirements, including formatting, file naming, and manuscript structure. We believe that now the manuscript fully adheres to the guidelines.

Attachment Submitted filename: Response to Reviewers.docx

# Peer review: Decision Letter 1

**DOI:** 10.1371/journal.pone.0361397.r003

**Authors:** Mitiku Badasa Moisa

25 Aug 2026

Dear Dr. Nazombe,

Thank you for submitting your manuscript to PLOS One. After careful consideration, we feel that it has merit but does not fully meet PLOS One’s publication criteria as it currently stands. Therefore, we invite you to submit a revised version of the manuscript that addresses the points raised during the review process.

Please submit your revised manuscript by Oct 09 2026 11:59PM. If you will need more time than this to complete your revisions, please reply to this message or contact the journal office at plosone@plos.org. When you're ready to submit your revision, log on to https://www.editorialmanager.com/pone/ and select the 'Submissions Needing Revision' folder to locate your manuscript file.

- A letter that responds to each point raised by the academic editor and reviewer(s). You should upload this letter as a separate file labeled 'Response to Reviewers'.
- A marked-up copy of your manuscript that highlights changes made to the original version. You should upload this as a separate file labeled 'Revised Manuscript with Track Changes'.
- An unmarked version of your revised paper without tracked changes. You should upload this as a separate file labeled 'Manuscript'.

If you would like to make changes to your financial disclosure, please include your updated statement in your cover letter. Guidelines for resubmitting your figure files are available below the reviewer comments at the end of this letter.

If applicable, we recommend that you deposit your laboratory protocols in protocols.io to enhance the reproducibility of your results. Protocols.io assigns your protocol its own identifier (DOI) so that it can be cited independently in the future. For instructions see: https://journals.plos.org/plosone/s/submission-guidelines#loc-laboratory-protocols. Additionally, PLOS One offers an option for publishing peer-reviewed Lab Protocol articles, which describe protocols hosted on protocols.io. Read more information on sharing protocols at https://plos.org/protocols?utm_medium=editorial-email&utm_source=authorletters&utm_campaign=protocols.

As the corresponding author, your ORCID iD is verified in the submission system and will appear in the published article. PLOS supports the use of ORCID, and we encourage all coauthors to register for an ORCID iD and use it as well. Please encourage your coauthors to verify their ORCID iD within the submission system before final acceptance, as unverified ORCID iDs will not appear in the published article. *Only*  the individual author can complete the verification step; PLOS staff *cannot*  verify ORCID iDs on behalf of authors.

We look forward to receiving your revised manuscript.

Kind regards,

Mitiku Badasa Moisa

Academic Editor

PLOS One

Journal Requirements:

If the reviewer comments include a recommendation to cite specific previously published works, please review and evaluate these publications to determine whether they are relevant and should be cited. There is no requirement to cite these works unless the editor has indicated otherwise.

[Note: HTML markup is below. Please do not edit.]

Reviewers' comments:

Reviewer's Responses to Questions

**Comments to the Author**

Reviewer #1: (No Response)

Reviewer #3: (No Response)

**********

2. Is the manuscript technically sound, and do the data support the conclusions?

Reviewer #1: Yes

Reviewer #3: Yes

**********

3. Has the statistical analysis been performed appropriately and rigorously?

Reviewer #1: Yes

Reviewer #3: (No Response)

**********

4. Have the authors made all data underlying the findings in their manuscript fully available?

The PLOS Data policy

Reviewer #1: (No Response)

Reviewer #3: (No Response)

**********

5. Is the manuscript presented in an intelligible fashion and written in standard English?

Reviewer #1: (No Response)

Reviewer #3: (No Response)

**********

Reviewer #1: The revision substantially improves the manuscript. The main first-round concerns (SRTM product identity, common control-point set, water-class sample size, DSM versus bare-earth interpretation, and addition of significance tests) have been addressed.

1. Vertical datum transformation is described but not reproducible. Please report the numerical value of Δv in the EGM96 alignment equation, whether it is a constant or a spatially varying offset, and the source used to obtain it.

2. Please state when the national geodetic control points were surveyed or last updated (even an approximate establishment period is sufficient).

3. Methods and the response letter describe independent-sample t-tests and Wilcoxon rank-sum tests, whereas Supplementary Table S3 is captioned as paired tests. Because the same 2,404 locations are used for all three DSMs, paired tests are the correct choice. Please make the Methods, Results, and Table S3 consistent. Relatedly, the national RMSE difference between SRTM (19.55 m) and NASADEM (19.99 m) is 0.44 m and should not be described as “substantially” lower.

4. The Abstract reports NASADEM RMSEs of 2.36–35.03 m. In Table 7, 2.36 m is the MAE for the Lower Shire Valley; the corresponding RMSE is 3.25 m. Please correct this.

5. High R² (0.9966–0.9971) should not be presented as evidence of vertical accuracy; it is expected given Malawi’s large elevation range. RMSE and MAE remain the appropriate accuracy metrics. Please add this caveat in the Results.

6. “(Huang & Yu, 2024)” remains as an author–year citation in the Introduction and is still missing from the numbered reference list. Please convert it to the journal’s numbered style.

Reviewer #3: Comments for “Vertical accuracy of ASTER, SRTM, and NASADEM Digital Surface Models across

Malawi’s diverse topography”

Dear Authors

Your manuscript is interesting and high potential for publication, but it needs major revision.

General comments

• The main finding of this study should be stated in abstract

• The resolution of all figures should be added

• All Tables should be Scientific Tables

• The necessity and innovation of the article should be presented to the introduction.

• It is suggested to compare the results of the present research with some similar studies which is done before. Refer add this findings

https://doi.org/10.1080/10298436.2025.2496335

https://doi.org/10.1016/j.triboint.2025.111348

https://doi.org/10.1007/s13131-025-2540-y

https://doi.org/10.1016/j.jhydrol.2025.133225

https://doi.org/10.1016/j.jhydrol.2025.132897

https://doi.org/10.1016/j.envsoft.2024.106138

https://doi.org/10.1016/j.rse.2025.115140

https://doi.org/10.1029/2025JC023214

https://doi.org/10.1016/j.jhydrol.2019.124516

https://doi.org/10.1002/nag.70323

• Much more explanations and interpretations must be added for the results, which are not enough and the discussions should be improved by comparing with previous studies.

https://doi.org/10.1016/j.enggeo.2026.108726

https://doi.org/10.1029/2026JE009657

10.1109/TGRS.2026.3654987

10.1130/B38406.1

https://doi.org/10.1016/j.ijmst.2026.01.008

https://doi.org/10.1016/j.ast.2026.113256

https://doi.org/10.3390/rs18142420

https://doi.org/10.12989/gae.2026.46.2.141

10.1007/s00603-025-05154-8

https://doi.org/10.1016/j.jvcir.2018.11.041

The major weakness of this study is that the debate/argument is not clearly articulated in the introduction. As a result, the paper’s contribution appears limited. I recommend that the author strengthen the theoretical discussion and explicitly develop the central debate/argument.

Please ensure that the conclusions section clearly highlights the scientific value added of the paper and/or the practical applicability of your findings, as previously indicated. Revise the conclusion to include more detail—specifically, emphasize your key contributions, discuss the study’s limitations, underscore the scientific value of the results, and outline implications for future research.

**********

what does this mean?). If published, this will include your full peer review and any attached files.

If you choose “no”, your identity will remain anonymous but your review may still be made public.

**Do you want your identity to be public for this peer review?**  For information about this choice, including consent withdrawal, please see our Privacy Policy

Reviewer #1: No

Reviewer #3: No

**********

[NOTE: If reviewer comments were submitted as an attachment file, they will be attached to this email and accessible via the submission site. Please log into your account, locate the manuscript record, and check for the action link "View Attachments". If this link does not appear, there are no attachment files.]

To ensure your figures meet our technical requirements, please review our figure guidelines: https://journals.plos.org/plosone/s/figures

You may also use PLOS’s free figure tool, NAAS, to help you prepare publication quality figures: https://journals.plos.org/plosone/s/figures#loc-tools-for-figure-preparation.

NAAS will assess whether your figures meet our technical requirements by comparing each figure against our figure specifications.

# Author comment: Author response to Decision Letter 2

**DOI:** 10.1371/journal.pone.0361397.r004

8 Oct 2026

Vertical datum transformation is described but not reproducible. Please report the numerical value of Δv in the EGM96 alignment equation, whether it is a constant or a spatially varying offset, and the source used to obtain it.

-Thank you for this comment. We have clarified the vertical datum transformation procedure in the revised manuscript. The original benchmark elevations were referenced to local Mean Sea Level (MSL), tied to the Beira tide gauge in Mozambique. A mean offset of +1.01 m between the local MSL datum and EGM2008 was applied to convert the benchmark elevations to EGM2008. The subsequent conversion from EGM2008 to EGM96 was performed using point-specific differences between the two geoid models obtained from a geoid calculator. The revised manuscript now clearly distinguishes these two transformation steps and explains how the final EGM96 reference elevations were obtained (Lines 220-234).

Please state when the national geodetic control points were surveyed or last updated (even an approximate establishment period is sufficient).

--Thank you for this comment. We have added information on the establishment period of the national geodetic control network. The Malawi geodetic control network was developed progressively between 1948 and 1974. The primary triangulation framework was established mainly during 1948-1953 and subsequent extensions and densification completed through 1974 (Lines 207-209).

Methods and the response letter describe independent-sample t-tests and Wilcoxon rank-sum tests, whereas Supplementary Table S3 is captioned as paired tests. Because the same 2,404 locations are used for all three DSMs, paired tests are the correct choice. Please make the Methods, Results, and Table S3 consistent. Relatedly, the national RMSE difference between SRTM (19.55 m) and NASADEM (19.99 m) is 0.44 m and should not be described as “substantially” lower.

--Thank you for pointing this out. We have revised the statistical analysis description throughout the manuscript to consistently use paired tests because the same 2,404 geodetic control locations were used to evaluate all three DSMs. The Methods and Results now refer to paired t-tests and Wilcoxon signed-rank tests (Lines 315-326). We also revised statements implying large practical differences between SRTM and NASADEM and now describe the observed national RMSE difference (0.44 m) as small despite statistical significance (Lines 495-498 and 643-644).

The Abstract reports NASADEM RMSEs of 2.36–35.03 m. In Table 7, 2.36 m is the MAE for the Lower Shire Valley; the corresponding RMSE is 3.25 m. Please correct this.

--We thank the reviewer for identifying this error. The Abstract has been corrected so that NASADEM RMSE values are now reported as 3.25 m (Line 33)

High R² (0.9966–0.9971) should not be presented as evidence of vertical accuracy; it is expected given Malawi’s large elevation range. RMSE and MAE remain the appropriate accuracy metrics. Please add this caveat in the Results.

--We thank the reviewer for this important clarification. We agree that very high R² values largely reflect the broad elevation range of Malawi rather than DSM vertical accuracy. A caveat has now been added to the Results section explaining that RMSE, MAE, and ME remain the primary measures of vertical accuracy (Lines 373-378).

“(Huang & Yu, 2024)” remains as an author–year citation in the Introduction and is still missing from the numbered reference list. Please convert it to the journal’s numbered style.

--We thank the reviewer for noting this oversight. The author-year citation has now been converted to the journal's numbered citation format and the full reference has been added to the reference list (lines 86 and 837).

The main finding of this study should be stated in abstract

--Thank you for this suggestion. The Abstract has been revised to explicitly state the principal finding of the study. In particular, we now emphasise that DSM accuracy is strongly terrain-dependent and that national-scale accuracy statistics can conceal substantial spatial variation. We also report the contrasting performance between the Lower Shire Valley and the Highlands.

The resolution of all figures should be added

--Thank you. All figures have been reviewed and prepared at publication-quality resolution using NAAS as recommended by the publisher.

All Tables should be Scientific Tables

--All tables have been reformatted using a consistent scientific table format.

The necessity and innovation of the article should be presented to the introduction.

--The Introduction has been substantially revised to clarify the research gap, necessity and innovation of the study. We now explain that national-scale accuracy statistics may conceal substantial spatial differences in DSM performance and explicitly state that the central argument of the study is that DSM suitability should be evaluated as a terrain-dependent property (lines 97-106).

It is suggested to compare the results of the present research with some similar studies which is done before. Refer add this findings

https://doi.org/10.1080/10298436.2025.2496335

https://doi.org/10.1016/j.triboint.2025.111348

https://doi.org/10.1007/s13131-025-2540-y

https://doi.org/10.1016/j.jhydrol.2025.133225

https://doi.org/10.1016/j.jhydrol.2025.132897

https://doi.org/10.1016/j.envsoft.2024.106138

https://doi.org/10.1016/j.rse.2025.115140

https://doi.org/10.1029/2025JC023214

https://doi.org/10.1016/j.jhydrol.2019.124516

https://doi.org/10.1002/nag.70323

---We carefully evaluated the suggested references and incorporated relevant papers where it directly supports the objectives of this study.

Much more explanations and interpretations must be added for the results, which are not enough and the discussions should be improved by comparing with previous studies.

https://doi.org/10.1016/j.enggeo.2026.108726

https://doi.org/10.1029/2026JE009657

10.1109/TGRS.2026.3654987

10.1130/B38406.1

https://doi.org/10.1016/j.ijmst.2026.01.008

https://doi.org/10.1016/j.ast.2026.113256

https://doi.org/10.3390/rs18142420

https://doi.org/10.12989/gae.2026.46.2.141

10.1007/s00603-025-05154-8

https://doi.org/10.1016/j.jvcir.2018.11.041

--We have substantially expanded both the Results and Discussion sections. The revised manuscript provides greater interpretation of the national-scale accuracy statistics, systematic elevation bias, terrain-dependent errors and land-cover effects (Lines 551-515, 517-519, 532-536, 593-597)

The major weakness of this study is that the debate/argument is not clearly articulated in the introduction. As a result, the paper’s contribution appears limited. I recommend that the author strengthen the theoretical discussion and explicitly develop the central debate/argument.

--We agree with this observation. The Introduction has been revised to explicitly articulate the central scientific argument of the study: that global DSM accuracy should not be treated as spatially uniform and that national-scale accuracy statistics may conceal substantial terrain-dependent uncertainty. The Discussion has also been reorganised to develop this argument using the observed differences among Malawi's physiographic regions. In particular, the small national RMSE difference between SRTM and NASADEM is contrasted with the much larger differences observed between lowland and highland environments (lines 641-653).

Please ensure that the conclusions section clearly highlights the scientific value added of the paper and/or the practical applicability of your findings, as previously indicated. Revise the conclusion to include more detail—specifically, emphasise your key contributions, discuss the study’s limitations, underscore the scientific value of the results, and outline implications for future research.

--The Conclusion has been substantially revised. It now clearly states the scientific contribution of demonstrating terrain-dependent DSM accuracy, summarises the major national and physiographic findings, discusses practical implications for hydrological, environmental, land-use and infrastructure applications, and identifies the main study limitations.

Attachment Submitted filename: Response_to_Reviewers_auresp_2.docx

# Editor report: Decision Letter 2

**DOI:** 10.1371/journal.pone.0361397.r005

**Authors:** Mitiku Badasa Moisa

11 Oct 2026

Vertical accuracy of ASTER, SRTM, and NASADEM Digital Surface Models across Malawi’s diverse topography

PONE-D-26-00523R2

Dear Dr. Nazombe,

We’re pleased to inform you that your manuscript has been judged scientifically suitable for publication and will be formally accepted for publication once it meets all outstanding technical requirements.

Within one week, you’ll receive an e-mail detailing the required amendments. When these have been addressed, you’ll receive a formal acceptance letter and your manuscript will be scheduled for publication.

An invoice will be generated when your article is formally accepted. Please note, if your institution has a publishing partnership with PLOS and your article meets the relevant criteria, all or part of your publication costs will be covered. Please make sure your user information is up-to-date by logging into Editorial Manager at Editorial Manager® and clicking the ‘Update My Information' link at the top of the page. For questions related to billing, please contact billing support.

If your institution or institutions have a press office, please notify them about your upcoming paper to help maximize its impact. If they’ll be preparing press materials, please inform our press team as soon as possible -- no later than 48 hours after receiving the formal acceptance. Your manuscript will remain under strict press embargo until 2 pm Eastern Time on the date of publication. For more information, please contact onepress@plos.org.

Kind regards,

Mitiku Badasa Moisa

Academic Editor

PLOS One

Additional Editor Comments (optional):

Dear Authors

Thank you for submitting the revised version of your manuscript. I have carefully reviewed your revisions against the reviewers' comments and suggestions. I am pleased to inform you that your paper has been accepted for publication. Congratulations on your fine work!

Reviewers' comments:

# Editor report: Acceptance letter

**DOI:** 10.1371/journal.pone.0361397.r006

**Authors:** Mitiku Badasa Moisa

PONE-D-26-00523R2

PLOS One

Dear Dr. Nazombe,

I'm pleased to inform you that your manuscript has been deemed suitable for publication in PLOS One. Congratulations! Your manuscript is now being handed over to our production team.

At this stage, our production department will prepare your paper for publication. This includes ensuring the following:

* All references, tables, and figures are properly cited

* All relevant supporting information is included in the manuscript submission,

* There are no issues that prevent the paper from being properly typeset

You will receive further instructions from the production team, including instructions on how to review your proof when it is ready. Please keep in mind that we are working through a large volume of accepted articles, so please give us a few days to review your paper and let you know the next and final steps.

Lastly, if your institution or institutions have a press office, please let them know about your upcoming paper now to help maximize its impact. If they'll be preparing press materials, please inform our press team within the next 48 hours. Your manuscript will remain under strict press embargo until 2 pm Eastern Time on the date of publication. For more information, please contact onepress@plos.org.

You will receive an invoice from PLOS for your publication fee after your manuscript has reached the completed accept phase. If you receive an email requesting payment before acceptance or for any other service, this may be a phishing scheme. Learn how to identify phishing emails and protect your accounts at https://explore.plos.org/phishing.

If we can help with anything else, please email us at customercare@plos.org.

Thank you for submitting your work to PLOS One and supporting open access.

Kind regards,

PLOS One Editorial Office Staff

on behalf of

Dr. Mitiku Badasa Moisa

Academic Editor

PLOS One
